# Green‐Chemistry‐Inspired Synthesis of Cyclobutane‐Based Hole‐Selective Materials for Highly Efficient Perovskite Solar Cells and Modules

**DOI:** 10.1002/anie.202113207

**Published:** 2021-12-16

**Authors:** Sarune Daskeviciute‐Geguziene, Yi Zhang, Kasparas Rakstys, Gediminas Kreiza, Sher Bahadar Khan, Hiroyuki Kanda, Sanghyun Paek, Maryte Daskeviciene, Egidijus Kamarauskas, Vygintas Jankauskas, Abdullah M. Asiri, Vytautas Getautis, Mohammad Khaja Nazeeruddin

**Affiliations:** ^1^ Department of Organic Chemistry Kaunas University of Technology Radvilenu pl. 19 Kaunas 50254 Lithuania; ^2^ Institute of Chemical Sciences and Engineering École Polytechnique Fédérale de Lausanne 1951 Sion Switzerland; ^3^ Institute of Photonics and Nanotechnology Vilnius University Saulėtekio al. 3 10257 Vilnius Lithuania; ^4^ Institute of Chemical Physics Vilnius University Saulėtekio al. 3 10257 Vilnius Lithuania; ^5^ Center of Excellence for Advanced Materials Research (CEAMR) King Abdulaziz University P.O. Box 80203 21589 Jeddah Saudi Arabia; ^6^ Department of Chemistry and Energy Engineering Sangmyung University Seoul 03016 Republic of Korea

**Keywords:** cyclobutane, green chemistry, hole-transporting materials, perovskites, solar cells

## Abstract

Hybrid lead halide perovskite solar cells (PSCs) have emerged as potential competitors to silicon‐based solar cells with an unprecedented increase in power conversion efficiency (PCE), nearing the breakthrough point toward commercialization. However, for hole‐transporting materials, it is generally acknowledged that complex structures often create issues such as increased costs and hazardous substances in the synthetic schemes, when translated from the laboratory to manufacture on a large scale. Here, we present cyclobutane‐based hole‐selective materials synthesized using simple and green‐chemistry inspired protocols in order to reduce costs and adverse environmental impact. A series of novel semiconductors with molecularly engineered side arms were successfully applied in perovskite solar cells. **V1366**‐based PSCs feature impressive efficiency of 21 %, along with long‐term operational stability under atmospheric environment. Most importantly, we also fabricated perovskite solar modules exhibiting a record efficiency over 19 % with an active area of 30.24 cm^2^.

## Graphical abstract

A molecularly engineered cyclobutane‐based hole‐transporting material synthesised using simple and green‐chemistry‐inspired protocols achieves an impressive efficiency of 21 % in perovskite solar cells and over 19 % in perovskite solar module with an active area of 30.24 cm^2^.

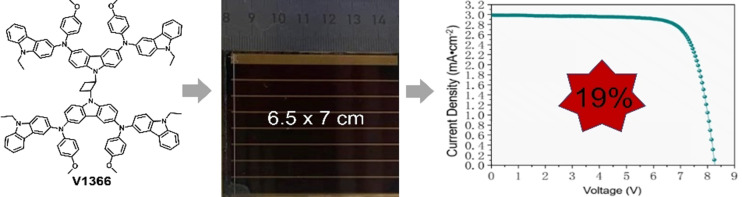

## Introduction

Although organic–inorganic perovskites have been known since the 19^th^ century, they have currently gained substantial attention in the field of photovoltaics and optoelectronics.^[1]^ Over the recent years, organic‐inorganic hybrid perovskite solar cells (PSCs) have been attracting massive worldwide attention due to their low cost and facile fabrication.^[2]^ Since 2009, when Miyasaka and co‐workers reported 3.8 % power conversion efficiency (PCE) of PSC,^[3]^ the performance of these photovoltaic devices has increased dramatically and currently PCE exceeds 25 %.^[4]^ Despite PSCs have skyrocketed in PCE, there are still several device issues that need to be resolved especially improving the long‐term stability.[^5^, ^6^, ^7^, ^8^, ^9^, ^10^] HTM is one of the quintessential components required for efficient and stable PSC devices. These materials are responsible for the transport of the photogenerated carriers from the absorber towards the electrode. HTMs should demonstrate sufficient charge transport properties, adequate energy levels, especially HOMO level, and good thermal stability. Despite significant research efforts devoted to developing new HTMs, these materials are still a weak spot in the PSC devices. In this context, small organic molecules are particularly appealing since they offer a wide range of structural modifications leading to the desired properties, and are easy to synthesize, purify, and process.[^11^, ^12^, ^13^, ^14^] Numerous approaches in the development of such HTMs including linear, star‐shaped, or spiro‐centered structures were reported in order to match the required hydrophobicity, energy levels, and the charge carrier mobility.[^15^, ^16^, ^17^, ^18^, ^19^, ^20^] To date, 2,2′,7,7′‐tetrakis‐(*N*,*N*‐di‐*p*‐methoxyphenylamine)‐9,9′‐spirobifluorene (spiro‐OMeTAD) dominates the field and despite its high price is routinely used as the gold standard for the research interests due to commercialization decades ago.^[21]^

As of the success of spiro‐OMeTAD, many research groups have been focused on spiro‐type compounds, expecting to improve the PCE with slight structural modifications.[^22^, ^23^] Several groups studied central 9,9′‐spirobifluorene‐linked HTMs including dimethylfluorenyl‐, ethylcarbazolyl‐, and fluorinated methoxyphenyl‐terminated examples recently reported by Seo,^[24]^ Chen,^[25]^ and Yang,^[26]^ respectively, as well as the development of new central spiro‐cored structures such as spiro[fluorene‐9,9′‐xanthene],[^27^, ^28^, ^29^, ^30^, ^31^] spirobisacridine,^[32]^ thiophene‐containing spiro cores[^33^, ^34^, ^35^] and other spiro‐type derivatives.[^36^, ^37^] However, the synthesis of such spiro‐type compounds typically requires a multi‐step reaction scheme involving low temperature (−78 °C), sensitive (*n*‐butyllithium), and aggressive (Br_2_) reagents resulting in a relative high material cost, consequently leading to a significant contribution to the total device cost and non‐negligible environmental impact.[^38^, ^39^, ^40^] The tedious synthesis and costly purification of HTMs may hamper large scale production and thereby could impede the overall commercial success of PSCs.

Therefore, the hunt is now on for new organic semiconductors that are prepared by simple, cost‐effective, and green chemistry without sacrificing the efficiency and would be easily scalable for a reasonable cost.[^41^, ^42^] In this sense, the utilization of a synthetic protocols that reduces or eliminates the use of hazardous substances is highly desirable. Moreover, simple product work up and purification may also significantly reduce the final synthesis cost and the environmental issues.[^43^, ^44^, ^45^] Recently, several research groups have focussed on tuning the structure by decreasing the number of synthetic steps, thus reducing the synthetic complexity, cost of materials and environmental impact.[^46^, ^47^]

Carbazole is known to be a promising core unit for molecular design since it can be substituted with a wide range of desired groups, allowing fine‐tuning of optical and electrochemical properties.^[48]^ Various carbazole‐containing scaffolds as electron donating units in the periphery were routinely used to tune the HOMO level and applied in PSCs, showing comparable photovoltaic performance.[^49^, ^50^, ^51^] This includes star‐shaped SGT series,[^52^, ^53^] benzodithiazole,^[54]^ bismethylenebenzene,[^55^, ^56^] bipyridine,^[57]^ pyrene‐based^[58]^ examples. Photodimerized carbazole is an attractive building block due to the simple, elegant and green synthesis and has been studied as excimer‐free and high hole carrier mobility material in early works.[^59^, ^60^, ^61^]

Herein, we disclose the development of novel HTMs, which comprises cyclobutane as a new structural core element for HTMs flanked by two differently substituted photodimerized carbazole arms in a branched fashion. The specific arrangement of carbazolyl groups onto cyclobutane core is also likely to facilitate the carrier transport process. Moreover, bulkiness and sterically hindered rigid *trans*‐configuration result in competition between the planarization and repulsive steric hindrance leading to a pseudo spiro type arrangement and diversified torsion angles. The effects of different peripheral carbazole substituents on various properties of newly synthesized molecules have been systematically investigated. Novel cyclobutane‐based HTMs have been successfully applied in PSCs, showing PCE up to 21 % and improved long‐term stability under atmospheric environment comparing to spiro‐OMeTAD. We also fabricated **V1366**‐based perovskite solar modules (6.5 cm × 7 cm) exhibiting a record efficiency over 19.0 % with an active area of 30.24 cm^2^ (corresponding to 16.78 % with an aperture area ≈34.36 cm^2^, a geometric fill factor of 88 %, the active area is used hereafter). Most importantly, to obtain novel HTMs we have applied protocols inspired by green chemistry, for the first time presenting that HTMs for PSCs could be synthesised eliminating the use of hazardous substances in order to reduce the adverse environmental impact without sacrificing the efficiency.

## Results and Discussion

**Figure 1:**
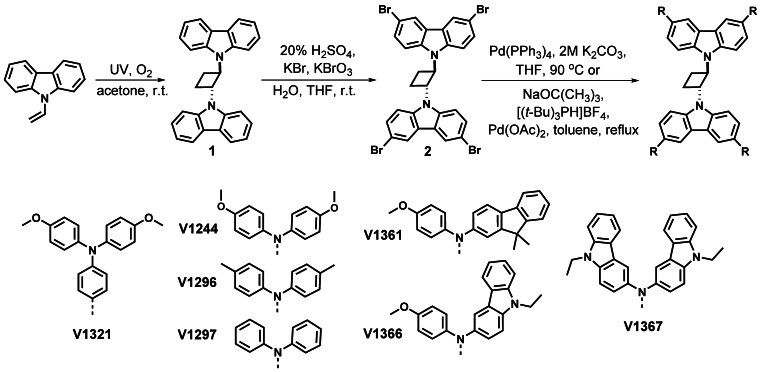
Synthetic route to novel hole‐transporting materials based on substituted cyclobutane.

The general synthesis procedure for the preparation of cyclobutane‐based HTMs is shown in Figure 1. The synthesis starts with the photochemical cyclodimerization of low‐cost commercially available 9‐vinyl carbazole. This step only required photoirradiation of starting material in green solvent acetone at ambient temperature. Next, *trans*‐1,2‐bis(9‐carbazolyl)cyclobutane (**1**) was brominated using an aqueous bromate‐bromide mixture as a green brominating agent to eliminate the use of aggressive bromine. To yield **V1321**, an aqueous/THF four‐fold Suzuki cross‐coupling procedure was applied. With this we demonstrate that all 3 synthetic steps required to obtain **V1321** were selected to reduce or eliminate the use of hazardous substances. Therefore, it could be classified as “green” HTM. To synthesise other HTMs presented in this work, **2** was reacted with the desired diarylamine‐based coupling partner under the standard Buchwald reaction conditions. Detailed synthetic protocols and full characterization of the compounds (NMR spectroscopy, mass spectrometry, and elemental analysis) are described in the Supporting Information.

Thermal gravimetric analysis (TGA) and differential scanning calorimetry (DSC) were used to determine thermal properties of the HTMs which are important to explore for processing temperatures and might affect the long‐term stability of the PSCs. TGA suggests that novel HTMs decompose in a range between 380–470 °C (Figure 3 a), far above the temperature for conventional device operation. From TGA results, there is a clear trend that higher molecular weight increases the thermal decomposition temperature (*T*_dec_). DSC measurements indicated that all new compounds are fully amorphous and have a glass transition temperature (*T*_g_) around 160 °C except **V1296**, which could exist in both crystalline and amorphous states as shown in Figure S9. Interestingly, all synthesized HTMs have higher *T*_g_ than spiro‐OMeTAD (124 °C) meaning that the cyclobutane‐based HTMs should have better morphological stability. In comparison, **V1367** has the highest *T*_g_ of 215 °C and should result in improved quality of the HTM layer. Moreover, microscope pictures revealed that among the series only **V1296** was prone for rapid formation of crystallization centers on the glass substrate, while other compounds resulted in fully transparent and amorphous films (Figure S10).

**Figure 2:**
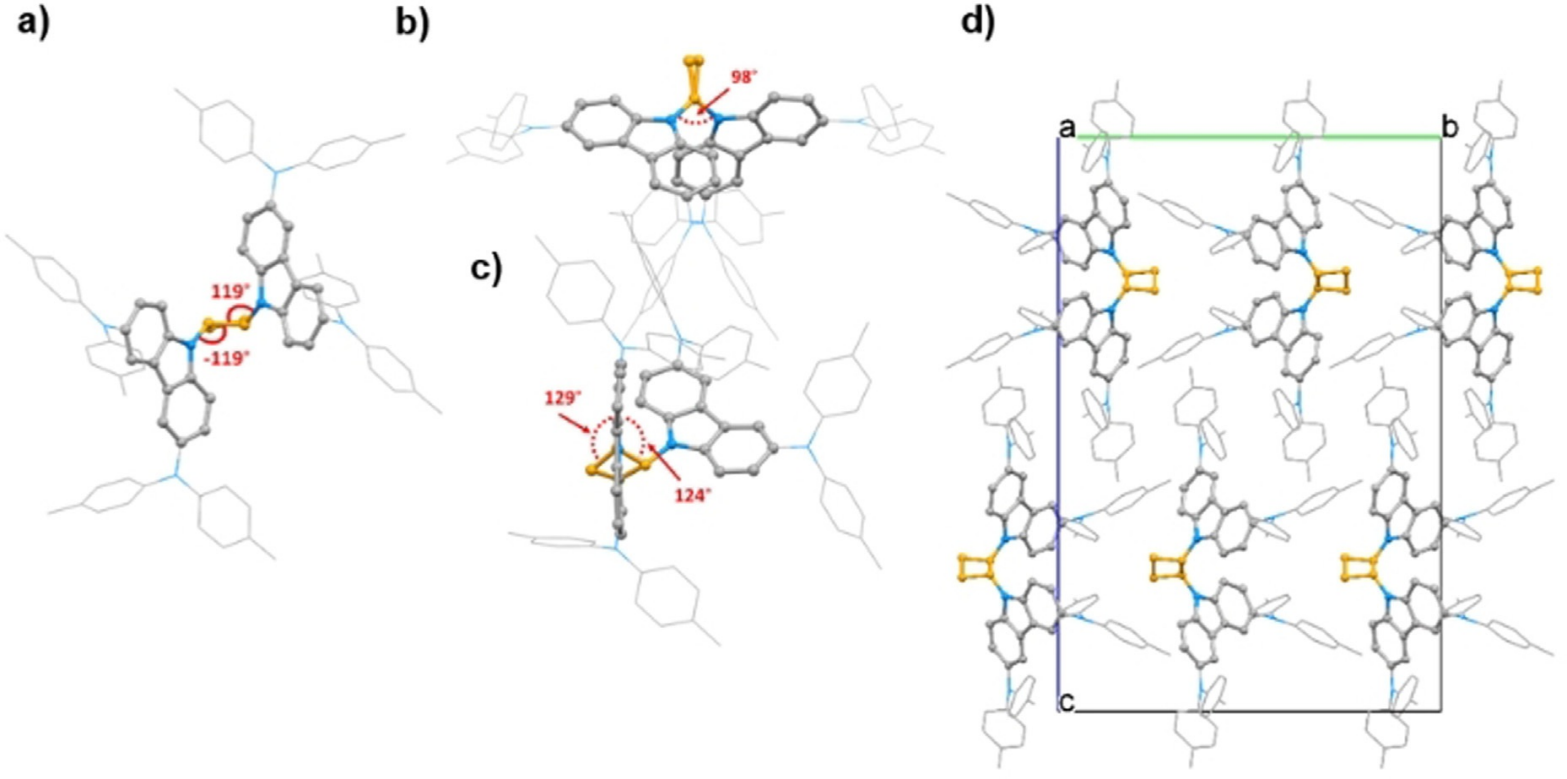
Molecular geometries of **V1296** obtained by X‐ray diffraction analysis with an indicated bond (a) and torsional angles (b and c). d) View down crystallographic *a*‐axis of the molecular packing model. For clarity, diphenylamine groups are shown as wireframe, the cyclobutane ring is coloured in orange, and hydrogen atoms are omitted.

**V1296** has been chosen as a model compound for X‐ray crystallography due to the highest crystallinity among the series to confirm the *trans*‐cyclobutane configuration and study the arrangement of carbazole substituents as the different electron donors around the carbazole should not affect the central core geometry and were ignored. **V1296** packs in orthorhombic spacegroup (*Pbcn*; No. 60) when grown by acetone vapor diffusion into chloroform solution. The following cell parameters were determined by single‐crystal X‐ray diffraction measurements: *a*=10.54500(10) Å, *b*=20.7442(4) Å, *c*=31.1322(4) Å, *α*=*β*=*γ*=90°, *V=*6810.09(17) Å3. The cell consists of four **V1296** molecules (*Z*=4) with an asymmetric unit equalling to half molecule (*Z′*=0.5). Molecular geometry and packing are visualized in Figure 2, whereas detailed crystallographic data is provided in Table S1. As visualized in Figure 2 a, two carbazole units were found to be attached to the central cyclobutane core with the same bond angles of 119° and −119°, respectively, indicating the *trans*‐configuration. Moreover, as shown in Figure 2 b, the dihedral angle between the cyclobutane‐connected carbazoles is measured to be 98° revealing a pseudo spiro conformation and being close to the dihedral angle between spiro‐connected fluorenes in spiro‐OMeTAD (90°).^[62]^ In addition, central cyclobutane ring was found to be not completely flat as demonstrated by different torsion angles shown in Figure 2 c.

**Figure 3:**
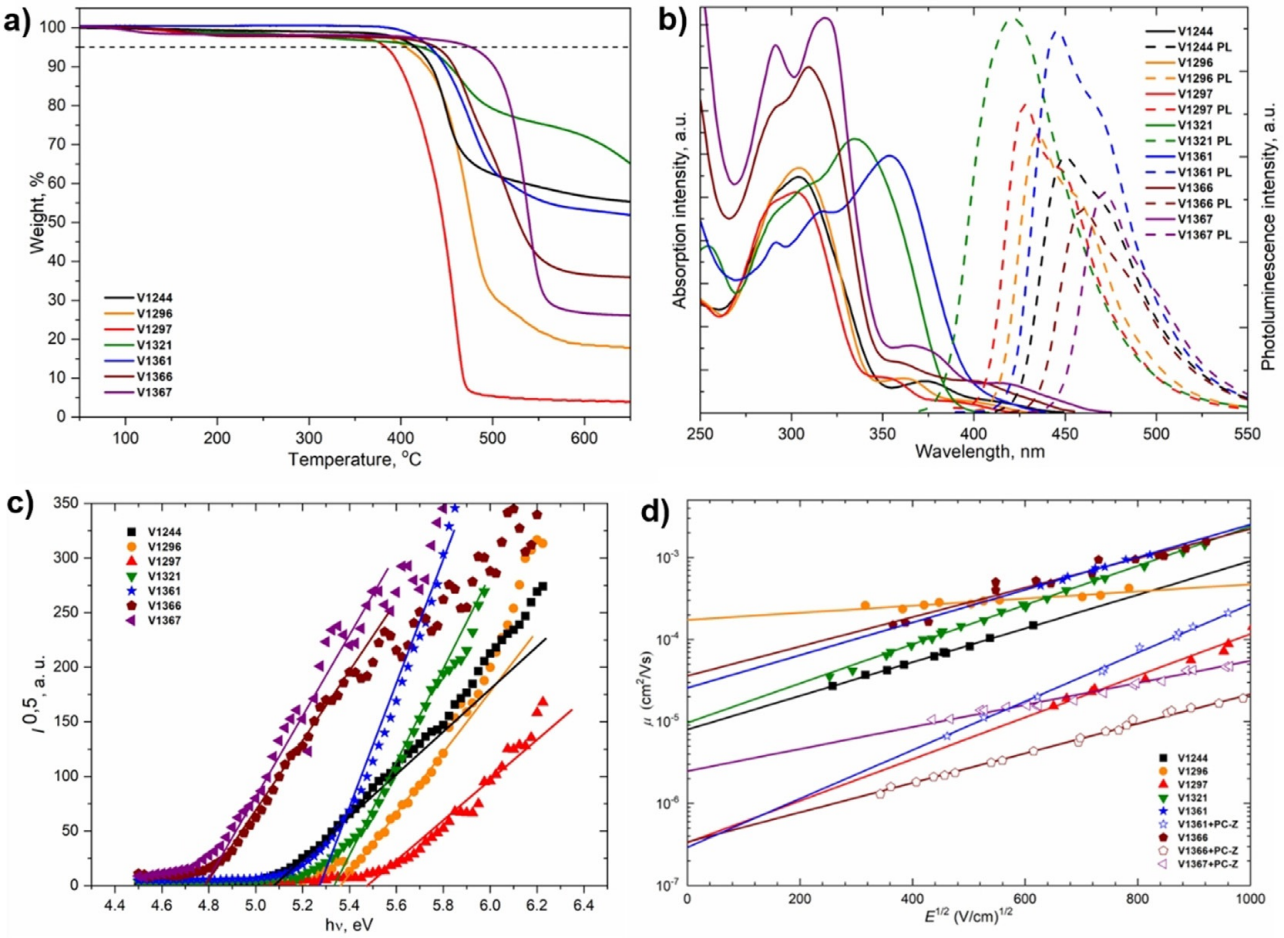
a) Thermogravimetric analysis (TGA) data (heating rate of 10 °C min^−1^, N_2_ atmosphere). b) UV/Vis absorption (solid line) and photoluminescence (dashed line) spectra of V‐series HTMs in THF solution (10^−4^ M). c) Photoemission spectra of the charge transporting layers measured in air. d) Electric field dependencies of the hole‐drift mobility in synthesized HTMs.

The ultraviolet‐visible absorption (UV/Vis) spectra in THF solutions of cyclobutane V‐series HTMs are shown in Figure 3 b. All new compounds have at least two major absorption peaks. The same absorption peak at 290 nm corresponds to localized π‐π* transitions arising from *trans*‐1,2‐bis(9‐carbazolyl)cyclobutane central scaffold, while absorption peaks at longer wavelengths arise from more intensive delocalization from the different conjugated substituents and are assigned to n‐π* transitions. The PL spectra revealed that significantly large Stokes shifts (100–150 nm) are observed for all molecules, therefore changes in the geometry of the molecules are expected upon excitation. The optical gaps (*E*_g_) were calculated from the intersection of absorption and photoluminescence spectra of thin films. They were found to be similar for all the compounds at around 3 eV (Figure S11).

The solid‐state ionization potentials (*I*_P_) of HTMs were determined using electron photoemission spectroscopy in air (PESA) of the thin films to assess the HOMO energy levels (Figure 3 c). *I*_P_ values of novel cyclobutanes were found to be lower than 5.7 eV, which equals to the valence band (VB) energy of the triple cation‐based perovskite, therefore efficient hole transfer from perovskite to the cathode should be ensured. Based on *E*_g_ and *I*_P_ values, we calculated the electron affinities (*E*_ea_) in the range of 1.9–2.5 eV. Importantly, calculated *E*_ea_ are smaller than the conduction band (CB) energy of the perovskite (−4.10 eV), ensuring the effective electron blocking from the perovskite to the electrode.

Xerographic time of flight (XTOF) measurements were used to determine the charge mobility of the V‐series layers. Dependences of hole drift mobility on electric field strength are shown in Figure 3 d. **V1296** exhibited the highest zero‐field hole drift mobility (*μ*_0_) among the series having the values of 1.7×10^−4^ cm^2^ Vs^−1^, outperforming that of spiro‐OMeTAD (*μ*_0_=1.3×10^−4^ cm^2^ Vs^−1^).^[63]^ The highest hole drift mobility of **V1296** could be explained due to its crystalline nature, however, the rapid crystallization in the film might result poor film forming properties and deteriorated PSC performance. **V1321**, **V1361**, and **V1366** showed one order of magnitude lower *μ*_0_ values. The thermal, optical, and photoelectrical properties of the cyclobutanes are summarized in Table 1.

**Table 1:** Thermal, optical, and photophysical properties of newly synthesized compounds.

Cmpd.	*T*_m_ [°C]^[a]^	*T*_g_ [°C]^[a]^	*T*_dec_ [°C]^[a]^	λ_abs_ [nm]^[b]^	λ_em_ [nm]^[b]^	*I*_P_ [eV]^[c]^	*E*_g_ [eV]^[d]^	*E*_ea_ [eV]^[e]^	*μ*_0_ [cm^2^ V^−1^ s^−1^]^[f]^
**V1244**	–	122	416	291, 303	450	5.07	2.91	2.12	7.9×10^−6^
**V1296**	320	159	406	291, 304	435	5.37	2.98	2.48	1.7×10^−4^
**V1297**	–	162	382	289, 302	427	5.48	3.05	2.43	3.3×10^−7^
**V1321**	–	148	421	291, 306, 334	420	5.34	3.13	2.21	1×10^−5^
**V1361**	–	157	432	291, 315, 354	445	5.28	2.93	2.35	2.5×10^−5^
**V1366**	–	173	439	291, 309	459	4.77	2.83	1.94	3.5×10^−5^
**V1367**	–	215	477	291,318	471	4.78	2.79	1.99	2.5×10^−6^

[a] Melting (*T*_m_), glass transition (*T*_g_), and decomposition (*T*_dec_) temperatures determined by DSC and TGA, respectively (10 °C min^−1^, N_2_ atmosphere). [b] Absorption and emission spectra were measured in THF solution (10^−4^  M). [c] Ionization energies of the films measured using PESA. [d] *E*_g_ estimated from the intersection of absorption and emission spectra of solid films. [e] *E*_ea_=*I*_P_−*E*_g_. [f] Mobility value at zero field strength.

**Figure 4:**
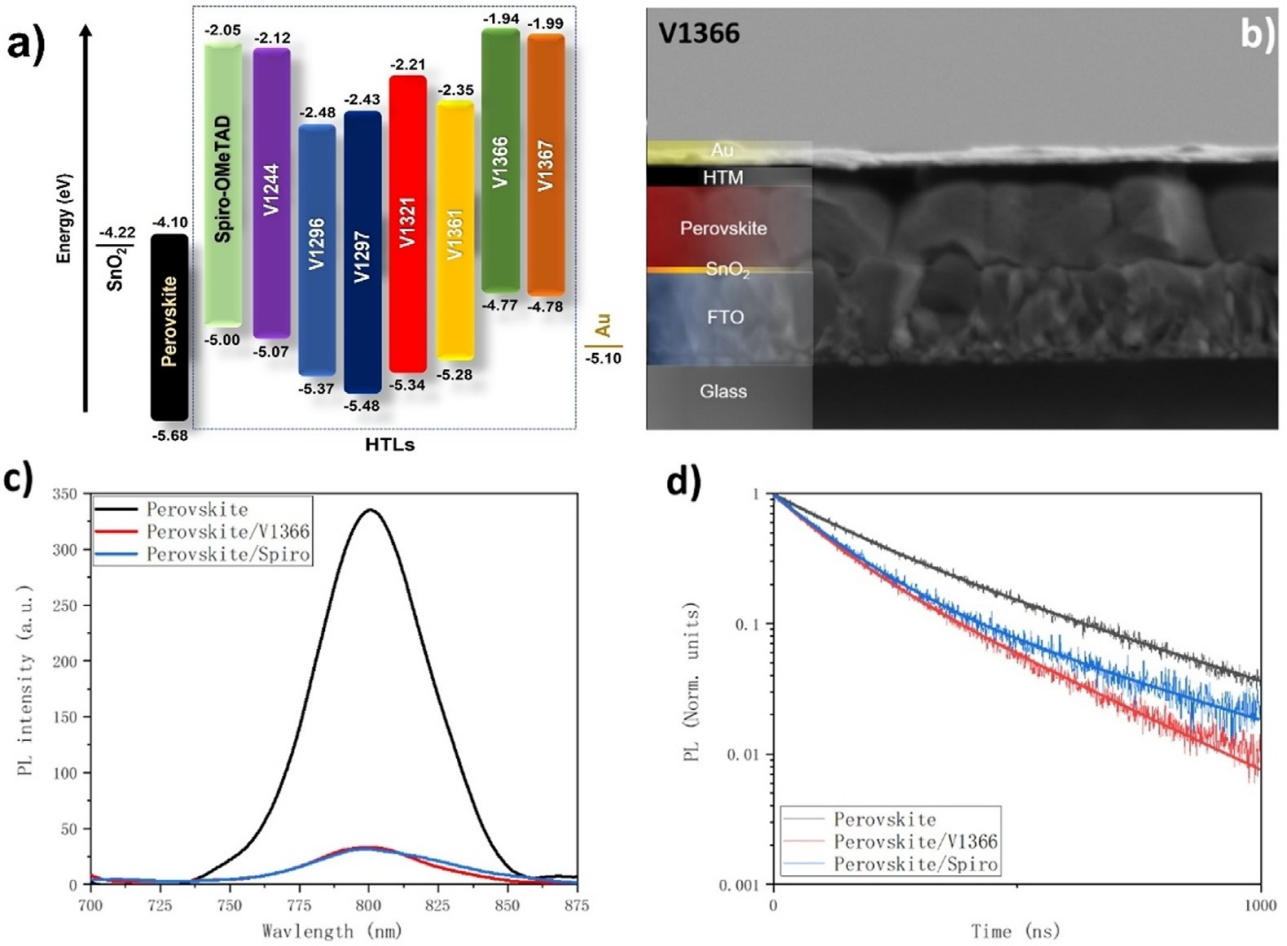
a) Schematic energy level diagram of the devices containing different HTLs. b) Cross‐sectional SEM image of the sample comprising FTO/SnO_2_/perovskite/**V1366**/Au layers. c) CW‐PL spectra (excitation: 480 nm) and d) PL lifetime of the perovskite thin films with or without spiro‐OMeTAD or **V1366**.

The schematic energy level diagram of the devices containing different HTLs is shown in Figure 4 a, and the detailed preparation process is described in the see the Supporting Information. Figure S12 and Figure 4 b represent the SEM of a cross‐sectional view of PSC devices with spiro‐OMeTAD and **V1366**, providing a direct view of the PSCs individual layers: FTO/SnO_2_/perovskite/HTM/Au. Thickness of the perovskite films are about 700 nm with 70 nm of Au layer. However, the thickness of the **V1366** layer is about 100 nm which is much thinner than that of the spiro‐OMeTAD layer (≈200 nm). This is due to the smaller molecule bulk and lower optimized concentration of the **V1366** solution. Due to the absence of carrier transport layer, as shown in Figure 4 c, higher continuous‐wave photoluminescence (CWPL) intensity is found in the perovskite thin film. When the HTM layers are covered, the CWPL intensities decrease sharply, and both HTM materials exhibit similar hole extraction capabilities. Also, the PL lifetime shown in Figure 4 d obtained from perovskite thin films was 286 ns (Table S2), which is almost 2 times longer than the results from perovskite/spiro‐OMeTAD and perovskite/**V1366** films.

**Figure 5:**
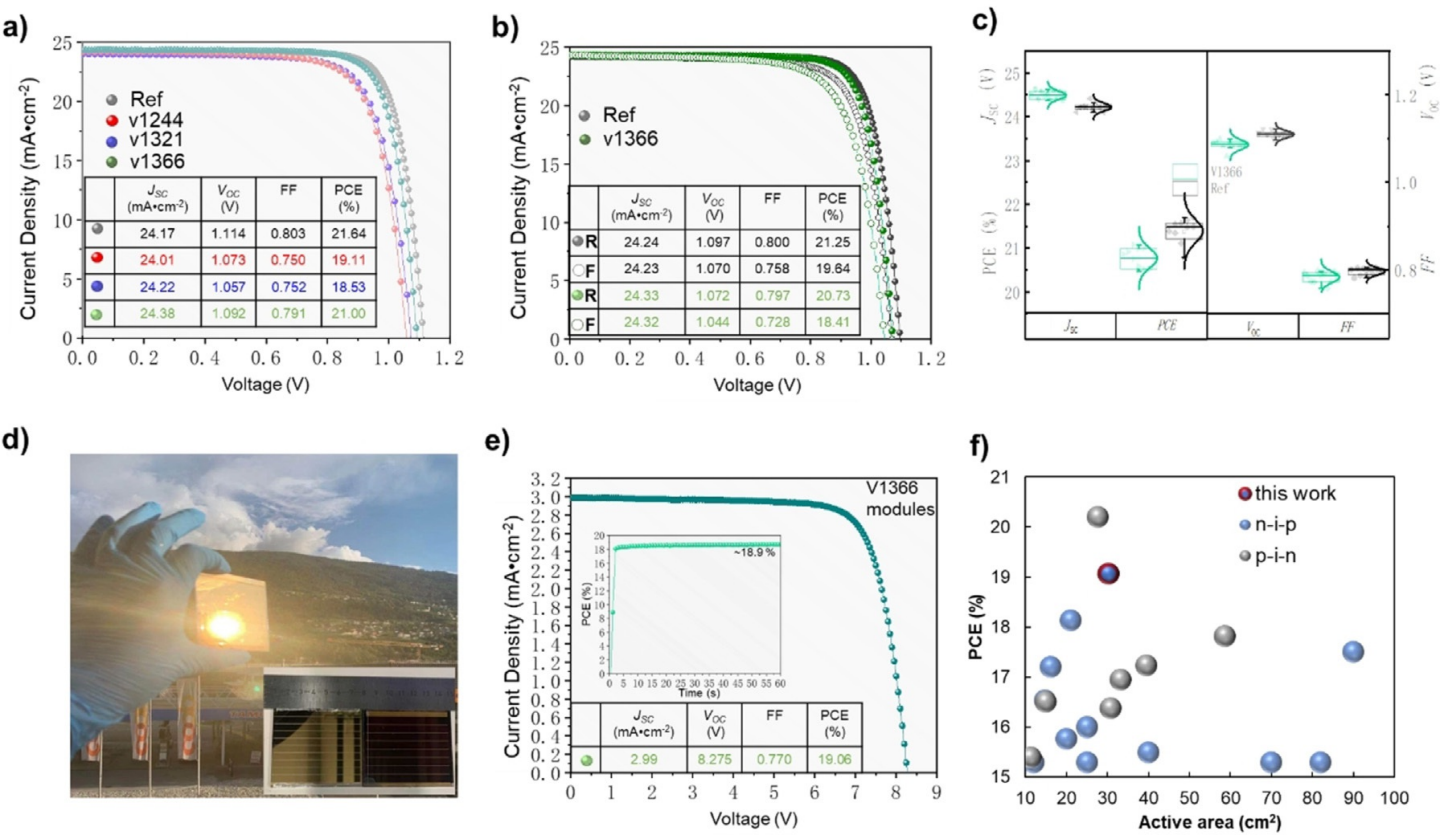
a) *J‐V* curves (reverse‐scan) of the PSCs based on **V1244**, **V1321**, and **V1366** as HTMs and spiro‐OMeTAD as the reference. b) *J‐V* hysteresis of spiro‐OMeTAD and **V1366**. c) Statistical deviation of the photovoltaic parameters for solar cells with **V1366** or spiro‐OMeTAD, respectively (10 different solar cells of each type). d) Photograph of the unsealed 6.5×7 cm solar module. e) *J*‐*V* curves of **V1366**‐based PSC module; the designated illumination area was estimated as 30.24 cm^2^; inset is the maximum‐power‐point power output of this module. f) Recently reported PCEs of perovskite solar modules with an active area of 10–100 cm^2^ and PCE over 15 % for both n‐i‐p and p‐i‐n architectures.

PSC devices using the different HTM materials were fabricated by sandwiching the perovskite thin films between an FTO/SnO_2_ anode and an HTM/Au cathode. Figure 5 a shows the typical current density—voltage (*J*‐*V*) curves (reverse scan) for the PSCs with spiro‐OMeTAD as a reference, **V1244**, **V1321**, and **V1366**, respectively. Devices having synthesised HTMs exhibit photoelectric conversion performance comparable to spiro‐OMeTAD, especially for the **V1366**, which showed even higher photocurrent. However, the devices with **V1296**, **V1297**, **V1361** and **V1367** as the HTMs exhibit relatively low PCE (Figure S14). Such deteriorated performance of **V1296** and **V1297** could be explained by quite deep HOMO levels, which could lead to the mismatch with the perovskite VB, while **V1367** has one of the lowest hole drift mobility among the series. On the other hand, the PCE of 21 % consisting of *J*_SC_ of 24.38 mA cm^−2^, a *V*_OC_ of 1.092 V, and an FF of 79.1 % was achieved for the **V1366**‐based device in comparison to 21.64 % for the spiro‐OMeTAD with *J*_SC_ of 24.17 mA cm^−2^, a *V*_OC_ of 1.114 V, and an FF of 80.3 %, showing that molecular engineering of side‐arms fully dictates the performance of the final device. The *J‐V* hysteresis of the best devices is shown in Figure 5 b. A similar hysteresis index of 1.08 for spiro‐OMeTAD and 1.12 for **V1366** device was found. A total of 20 solar cells in two groups were fabricated under the same conditions with spiro‐OMeTAD or **V1366** as the HTM. Figure 5 c demonstrates the statistical distribution of all four photovoltaic parameters of the two groups of solar cells to show the reproducibility of each condition. All the photovoltaic parameters of them show a similar half‐width, which means the reproducibility of **V1366**‐based devices is comparable to that of spiro‐OMeTAD (Table S3). As shown in the Figure S13, we observe an iodine/lead atoms ratio (1.84) in the spiro‐OMeTAD/perovskite layer while a constant iodine/lead atoms ratio (1.42) is observed in the **V1366**/perovskite layer, which means more iodine should be diffused into the spiro‐OMeTAD between the interface of perovskite thin films and HTL.

Another issue of importance to commercialization is the large‐scale production when translating from laboratory to manufacturing scale. To characterize the upscaling performance of the new HTM, we fabricated **V1366**‐based perovskite modules sized of 6.5×7 cm as shown in Figure 5 d. The module exhibited a PCE of 19.06 % with *J*_SC_ of 2.99 mA cm^−2^, a *V*_OC_ of 8.275 V, and an FF of 77 % as shown in Figure 5 e. And the PCE of the module stabilizes at ≈19 %, consistent with the reverse‐scan *J‐V* curve. To the best of our knowledge, the PCE value over 19 % is the highest PCE ever reported for non‐spiro‐OMeTAD based perovskite module. This is also illustrated in Figure 5 f and Table S4, to facilitate the comparison of the device itself, we summarized the recent reports with photovoltaic performance and device structure of perovskite solar modules prepared by different methods with an active area of 10–100 cm^2^ and PCE over 15 % including both n‐i‐p and p‐i‐n architectures. In addition, from the broader view, the highly efficient module using **V1366** not only shows one of the state‐of‐the‐art performances for both n‐i‐p and p‐i‐n architectures but also clearly shows the proficient scalability and combined advantages of green‐chemistry approach developed **V1366** HTM.

**Figure 6:**
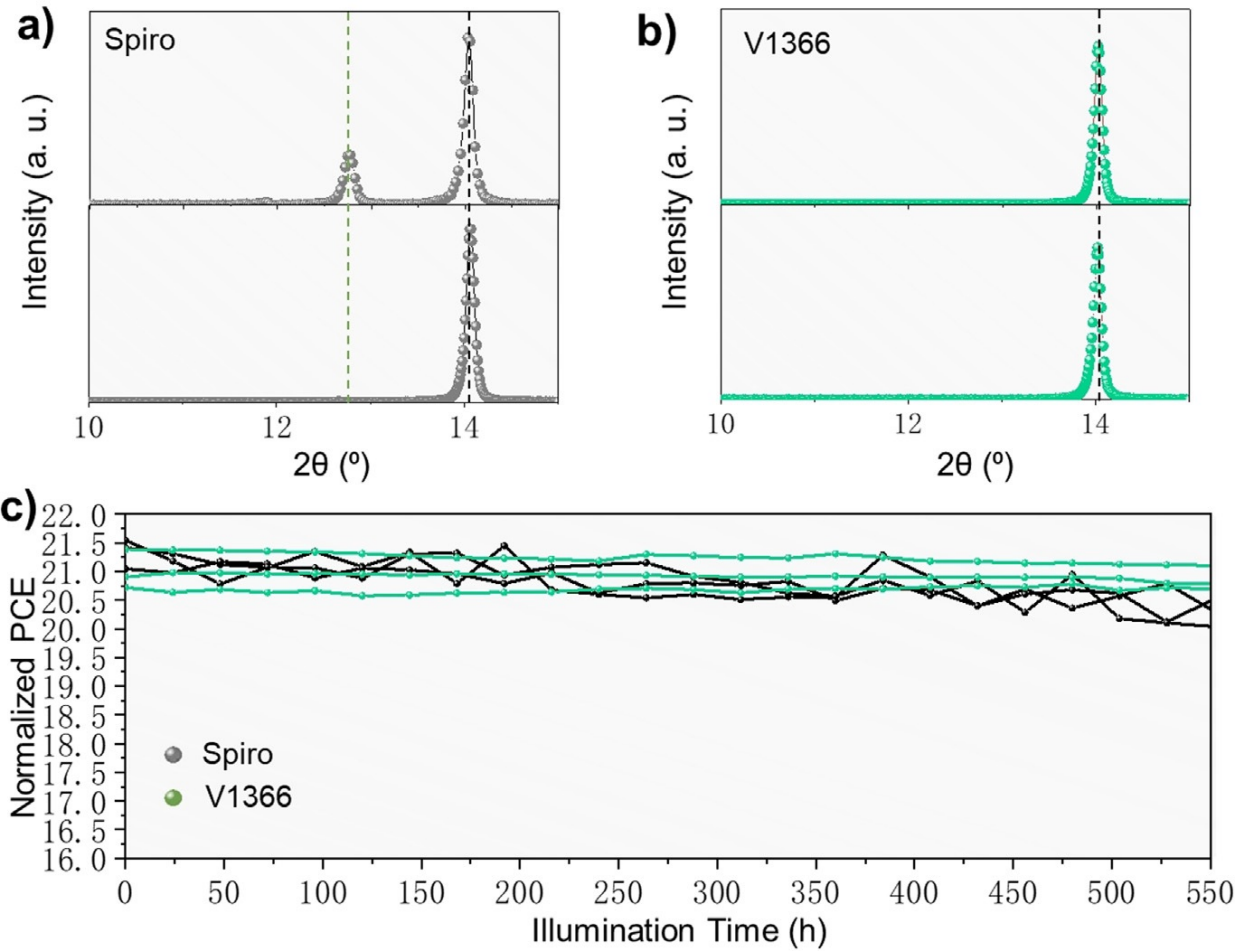
Long‐term stability of the perovskite thin films and devices. Representative XRD patterns of the perovskite thin films with different HTMs before and after 5 h heating (85 °C) under ambient conditions (60 % RH): a) spiro‐OMeTAD, b) **V1366**. The green and black dashed lines indicate the peak positions of the 001 reflection of the PbI_2_ crystal phase and the 110 reflection of the perovskite phase, respectively. c) Long‐term stability of PSC devices (stored in the drawer, tested every 24 hours, 15 %–20 % RH, RT) made with spiro‐OMeTAD and **V1366**.

In addition to the PCE, the chemical stability was also evaluated showing the increased stability of **V1366**‐based device, which represents another important advantage of the new HTM. Figure 6 a, b shows representative XRD patterns of the perovskite thin films with spiro‐OMeTAD and **V1366** before and after 5 h heating (85 °C) under ambient conditions (60 % RH). While the reference perovskite thin‐film shows decomposition to PbI_2_ after storage, the **V1366**‐based film maintains its good phase purity. It is most likely that the **V1366** slows the moisture ingression kinetics, due to denser layer structure and less doping. The long‐term device stability is tested every 24 hours by operating the three PSCs per condition. While the reference device shows a decrease of more than 5 % in PCE after 550 h, almost no PCE loss is observed for the **V1366**‐based PSC, demonstrating the enhanced device stability.

## Conclusion

Drawing the results together, we report the synthesis and a systematic study of the cyclobutane‐based hole‐transporting materials that are synthesized by simple and “green” chemistry. The impact of the different side‐arm fragments onto cyclobutane central core was revealed through the optical, electrochemical, photophysical, and photovoltaic measurements. It was found that cyclobutane fragment increases the glass transition temperature of final HTMs being more amorphous and morphologically stable. Additionally, hole drift mobility values of cyclobutane‐centered HTMs up to 10^−4^ cm^2^ Vs^−1^ order of magnitude, have been reached which outperforms spiro‐OMeTAD. The most efficient perovskite devices contained **V1366** reaching the PCE of 21 % and excellent long‐term stability. Most importantly, we fabricated perovskite solar modules exhibiting a record efficiency over 19 % with an active area of 30.24 cm^2^. The results of this study cover the main requirements for the successful implementation of perovskite solar cell technology.

## Conflict of interest

The authors declare no conflict of interest.

## Supporting information

As a service to our authors and readers, this journal provides supporting information supplied by the authors. Such materials are peer reviewed and may be re‐organized for online delivery, but are not copy‐edited or typeset. Technical support issues arising from supporting information (other than missing files) should be addressed to the authors.

Supporting Information
