# Supplementary material for: Green‐Chemistry‐Inspired Synthesis of Cyclobutane‐Based Hole‐Selective Materials for Highly Efficient Perovskite Solar Cells and Modules
Source: Angew Chem Int Ed Engl. 2021 Dec 16;61(5):e202113207. doi: 10.1002/anie.202113207 (PMC9299821; doi:10.1002/anie.202113207)
Supplement: Supplementary file 1 — Supporting Information [file ANIE-61-0-s001.pdf]

Angewandte Chemie
Eine Zeitschrift der Gesellschaft Deutscher Chemiker

Supporting Information

Green-Chemistry-Inspired Synthesis of Cyclobutane-Based Hole-Selective Materials for Highly Efficient Perovskite Solar Cells and Modules

Sarune Daskeviciute-Geguziene, Yi Zhang, Kasparas Rakstys, Gediminas Kreiza, Sher Bahadar Khan, Hiroyuki Kanda, Sanghyun Paek, Maryte Daskeviciene, Egidijus Kamarauskas, Vygintas Jankauskas, Abdullah M. Asiri, Vytautas Getautis,* and Mohammad Khaja Nazeeruddin**

anie_202113207_sm_miscellaneous_information.pdf

Supporting Information

Experimental section

Chemicals required for the synthesis were purchased from Sigma-Aldrich, TCI Europe, and Fluorochem and were used as received without additional purification. ^{1}H NMR spectra were recorded at 400 MHz on a Bruker Avance III spectrometer with a 5 mm double resonance broad band BBO z-gradient room temperature probe, ^{13}C NMR spectra were collected using the same instrument at 101 MHz. The chemical shifts, expressed in ppm, were relative to tetramethylsilane (TMS). All the NMR experiments were performed at 25 °C. Reactions were monitored by thin-layer chromatography on ALUGRAM SIL G/UV254 plates and developed with UV light. Silica gel (grade 9385, 230–400 mesh, 60 Å, Aldrich) was used for column chromatography. Elemental analysis was performed with an Exeter Analytical CE-440 elemental analyser, Model 440 C/H/N/.

Detailed synthetic procedures

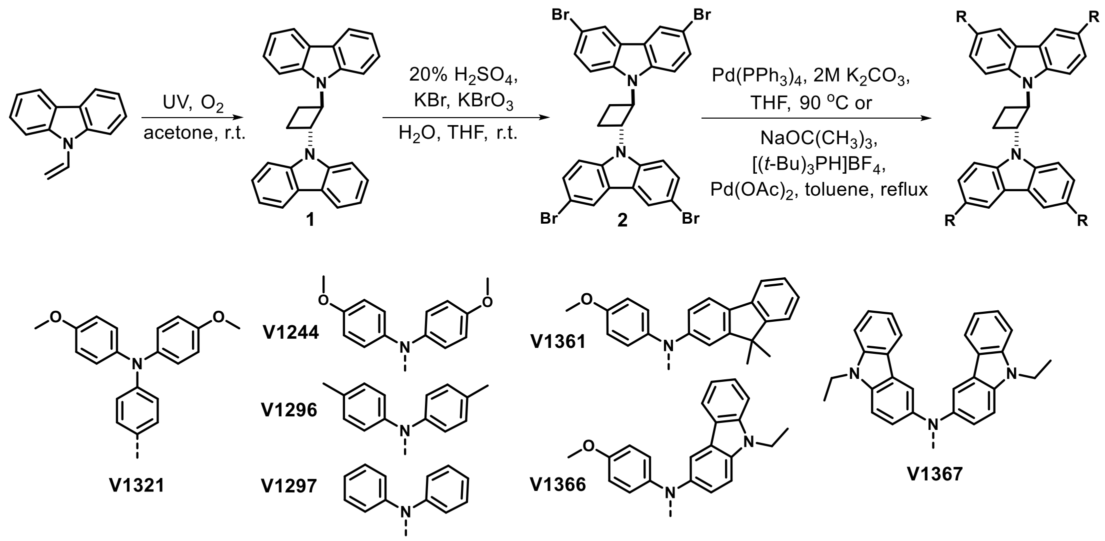

Figure S1. Synthesis scheme for final HTMs.

1,2-di(9*H*-carbazol-9-yl)cyclobutane (1)

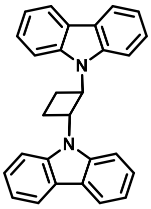

A solution of 9-vinylcarbazole (12 g, 62 mmol) in acetone (125 mL) was irradiated (GR.E. 125W helios italquartz) for 15 hours at room temperature. Air was bubbled through the solution continuously. The precipitated product was filtered and recrystallized from acetone to give pale creamy crystals. (8.5 g, 70.8%). ^{1}H NMR (400 MHz, THF-*d*$_6$) δ 8.02 (d, *J* = 8.0 Hz, 4H), 7.72 (d, *J* = 8.0 Hz, 4H), 7.34 (t, *J* = 7.6 Hz, 4H), 7.13 (t, *J* = 7.6 Hz, 4H), 6.53 – 6.29 (m, 2H), 3.22 – 2.99 (m, 2H), 2.80 – 2.63 (m, 2H). ^{13}C NMR (101 MHz, THF) 138.27, 123.59, 121.69, 118.15, 117.15, 107.88, 52.48, 18.59.

1,2-bis(3,6-dibromo-9*H*-carbazol-9-yl)cyclobutane (2)

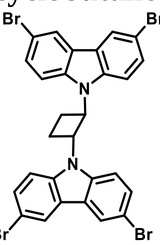

Compound **(1)** (1.9 g, 4.9 mmol) was dissolved in THF (50 mL). Afterwards, 20% H_2SO_4 (50 mL) solution was added, following by dropwise addition (10mL/min) of KBr and $KBrO_3$ solution (69 mL H_2O, KBr 4.1 g, $KBrO_3$ 1.15 g) and stirred at room temperature for 72 hours. The precipitate was collected by filtration, washed with water and hot methanol for three times to give **2** as white crystals. (3.1 g, 88.6%). ^{1}H NMR (400 MHz, THF-*d*$_6$) δ 8.26 (s, 4H), 7.65 (d, *J* = 8.8 Hz, 4H), 7.50 (d, *J* = 8.8 Hz, 4H), 6.41 – 6.13 (m, 2H), 3.14 – 2.96 (m, 2H), 2.85 – 2.64 (m, 2H). ^{13}C NMR (101 MHz, THF) δ 139.05, 129.02, 124.33, 123.45, 112.47, 111.59, 54.51, 20.75.

1,2-bis[3,6-bis(4,4'-dimethoxy)diphenylamino-9*H*-carbazol-9-yl]cyclobutane (V1244)

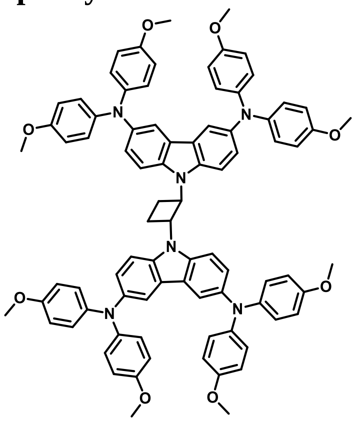

A solution of compound **2** (0.5 g, 0.7 mmol, 1 eq) and 4,4'-dimethoxydiphenylamine (0.98 g, 4.3 mmol, 6 eq) in anhydrous toluene (7 mL) was purged with argon for 30 minutes. Afterwards, palladium (II) acetate (0.02 eq), tri-*tert*-butylphosphonium tetrafluoroborate (0.027 eq) and sodium *tert*-butoxide (6 eq) were added and the solution was refluxed under argon atmosphere for 5 hours. After cooling to room temperature, reaction mixture was filtered through celite, extracted with ethylacetate and distilled water. The organic layer was dried over anhydrous Na_2SO_4, filtered and solvent evaporated. The crude product was purified by column chromatography using 3:9.5 v/v THF/*n*-hexane as an eluent. The obtained product was dissolved in acetone and precipitated into 15 times excess of ethanol. The precipitate was filtered off and washed with ethanol to collect **V1244** as a pale green solid. (0.52 g, 56.3%). ^{1}H NMR (400 MHz, THF-d_6) δ 7.66 – 7.51 (m, 8H), 7.08 (d, J = 8.8, 1.7 Hz, 4H), 6.88 (d, J = 8.8 Hz, 16H), 6.71 (d, J = 8.8 Hz, 16H), 6.34 – 6.18 (m, 2H), 3.69 (s, 24H), 3.03 – 2.91 (m, 2H), 2.70 – 2.60 (m, 2H). ^{13}C NMR (101 MHz, THF) δ 154.95, 142.47, 141.24, 137.03, 124.27, 124.13, 123.92, 116.39, 114.17, 110.55, 54.75, 54.54, 20.62. Anal. calcd for $C_{84}H_{74}N_6O_8$: C, 77.88; H, 5.76; N, 6.49; found: C, 77.97; H, 5.72; N, 6.41. $C_{84}H_{74}N_6O_8[M^+]$ exact mass = 1294.56, MS (ESI) = 1294.97.

1,2-bis[3,6-bis(4,4'-dimethyl)diphenylamino-9*H*-carbazol-9-yl]cyclobutane (V1296)

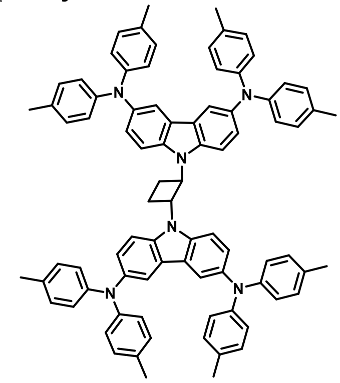

A solution of compound **2** (0.5 g, 0.7 mmol, 1 eq) and 4,4'-dimethyldiphenylamine (0.84 g, 4.3 mmol, 6 eq) in anhydrous toluene (7 mL) was purged with argon for 30 minutes. Afterwards, palladium (II) acetate (0.02 eq), tri-*tert*-butylphosphonium tetrafluoroborate (0.027 eq) and sodium *tert*-butoxide (6 eq) were added and the solution was refluxed under argon atmosphere for 22 hours. After cooling to room temperature, reaction mixture was filtered through celite, extracted with ethylacetate and distilled water. The organic layer was dried over anhydrous Na_2SO_4, filtered and solvent evaporated. The crude product was recrystallized from ethanol/toluene 1:1 to give **V1296** as pale green crystals. (0.46 g, 55.4%). ^{1}H NMR (400 MHz, THF-d_6) δ 7.67 (s, 4H), 7.66 (d, J = 8.8 Hz, 4H), 7.12 (d, J = 8.8 Hz, 4H), 6.93 (d, J = 8.4 Hz, 16H), 6.85 (d, J = 8.4 Hz, 16H), 6.39 – 6.25 (m, 2H), 3.09 – 2.92 (m,

2H), 2.79 – 2.59 (m, 2H), 2.22 (s, 24H). ^{13}C NMR (101 MHz, THF) δ 144.59, 138.67, 135.70, 128.59, 127.45, 123.16, 122.52, 120.71, 116.07, 108.91, 52.89, 18.84, 17.92. Anal. calcd for $C_{84}H_{74}N_6$: C, 86.41; H, 6.39; N, 7.20; found: C, 86.24; H, 6.45; N, 7.31. $C_{84}H_{74}N_6$[M$^+$] exact mass = 1166.60, MS (ESI) = 1167.06.

1,2-bis(3,6-bisdiphenylamino-9*H*-carbazol-9-yl)cyclobutane (V1297)

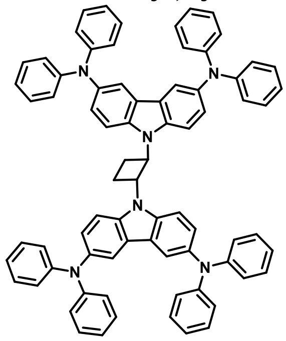

A solution of compound **2** (0.5 g, 0.7 mmol, 1 eq) and diphenylamine (0.72 g, 4.3 mmol, 6 eq) in anhydrous toluene (7 mL) was purged with argon for 30 minutes. Afterwards, palladium (II) acetate (0.02 eq), tri-*tert*-butylphosphonium tetrafluoroborate (0.027 eq) and sodium *tert*-butoxide (6 eq) were added and the solution was refluxed under argon atmosphere for 27 hours. After cooling to room temperature, reaction mixture was filtered through celite, extracted with ethylacetate and distilled water. The organic layer was dried over anhydrous Na_2SO_4, filtered and solvent evaporated. The crude product was purified by column chromatography using 1:9 v/v THF/*n*-hexane as an eluent. The obtained product was dissolved in THF and precipitated into 15 times excess of *n*-hexane. The precipitate was filtered off and washed with hexane to collect **V1297** as a pale green solid. (0.44 g, 58.7%). ^{1}H NMR (400 MHz, DMSO-d_6) δ 7.89 (d, *J* = 9.2 Hz, 4H), 7.83 (d, *J* = 2.0 Hz, 4H), 7.27 – 7.05 (m, 20H), 6.97 – 6.79 (m, 24H), 6.39 – 6.24 (m, 2H), 2.93 – 2.75 (m, 2H), 2.70 – 2.55 (m, 2H). ^{13}C NMR (101 MHz, DMSO) δ 148.42, 139.76, 138.02, 129.65, 126.33, 124.09, 122.46, 122.02, 119.67, 112.27, 54.24, 21.65. Anal. calcd for $C_{76}H_{58}N_6$: C, 86.50; H, 5.54; N, 7.96; found: C, 86.65; H, 5.50; N, 7.85. $C_{76}H_{58}N_6$[M$^+$] exact mass = 1054.47, MS (ESI) = 1054.90.

1,2-bis|3,6-bis{4-[*N,N*-bis(4-methoxyfenil)amino]fenil}-9*H*-carbazol-9-yl|cyclobutane (V1321)

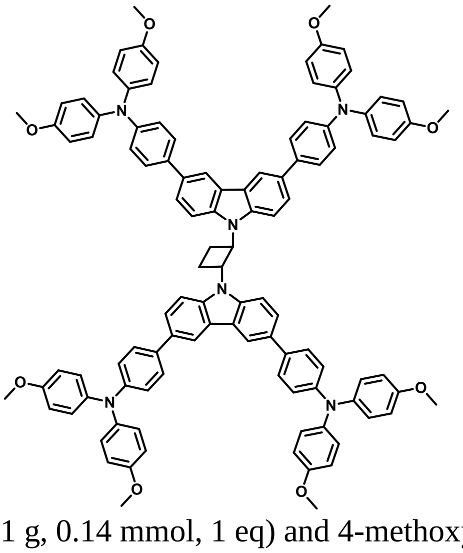

A solution of compound **2** (0.1 g, 0.14 mmol, 1 eq) and 4-methoxy-*N*-(4-methoxyphenyl)-*N*-(4-(4,4,5,5-tetramethyl-1,3,2-dioxaborolan-2-yl)phenyl)aniline (0.61 g, 1.4 mmol, 10 eq) in anhydrous THF (10 mL) was purged with argon for 10 minutes. Afterwards,

tetrakis(triphenylphosphine)-palladium(0) (0.115 eq) and 2M K_2CO_3 (4 mL) were added and the solution was heated for 3 hours at 90 ºC. After cooling to room temperature, reaction mixture was filtered through celite, extracted with ethylacetate and distilled water. The organic layer was dried over anhydrous Na_2SO_4, filtered and solvent evaporated. The crude product was purified by column chromatography using 4:8.5 v/v THF/*n*-hexane as an eluent. The obtained product was dissolved in THF into 15 times excess of *n*-hexane. The precipitate was filtered off and washed with hexane to collect **V1321** as a pale yellow - green solid. (0.16 g, 70.2%). ^{1}H NMR (400 MHz, THF-*d*$_6$) δ 8.34 (s, 4H), 7.76 (d, *J* = 8.8 Hz, 4H), 7.59 (d, *J* = 8.8 Hz, 4H), 7.51 (d, *J* = 8.6 Hz, 8H), 7.02 (d, *J* = 8.8 Hz, 16H), 6.97 (d, *J* = 8.6 Hz, 8H), 6.82 (d, *J* = 8.8 Hz, 16H), 6.50 – 6.35 (m, 2H), 3.74 (s, 24H), 3.19 – 3.02 (m, 2H), 2.86 – 2.68 (m, 2H). ^{13}C NMR (101 MHz, THF) δ 154.17, 145.70, 139.26, 137.80, 132.26, 130.62, 125.34, 124.20, 122.65, 122.48, 119.37, 115.94, 112.55, 108.21, 52.85, 52.74, 18.86. Anal. calcd for $C_{108}H_{90}N_6O_8$: C, 81.08; H, 5.67; N, 5.25; found: C, 81.35; H, 5.54; N, 5.23. $C_{108}H_{90}N_6O_8[M^+]$ exact mass = 1598.68, MS (ESI) = 1599.45.

N-(4-methoxyphenyl)-9,9-dimethyl-9*H*-fluoren-2-amine (3)

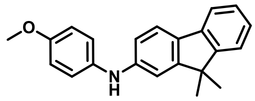

Anhydrous dioxane (12 mL) with few drops of distilled water (0.02 eq) was purged with argon for 20 minutes. After that, the temperature was raised to 80 °C, palladium (II) acetate (0.005 eq) and XPhos (0.015 eq) were added. The mixture was stirred for 1.5 minutes and temperature was raised to 110 ºC. 2-bromo-9,9-dimethylfluorene (2.7 g, 9.9 mmol, 1 eq), *p*-Anisidine (1.5 g, 11.9 mmol, 1.2 eq) and sodium *tert*-butoxide (1.4 eq) were added and stirred for 1 hour. After cooling to room temperature, reaction mixture was extracted with ethyl acetate and distilled water. The organic layer was dried over anhydrous Na_2SO_4, filtered and the solvent evaporated. The crude product was purified by column chromatography using 1:12.5 v/v THF/*n*-hexane as an eluent. Pale brown crystals were collected as a final product. (2.45 g, 78.7 %). ^{1}H NMR (400 MHz, DMSO-*d*$_6$) δ 8.01 (s, 1H), 7.68 – 7.53 (m, 2H), 7.44 (d, *J* = 7.6 Hz, 1H), 7.25 (t, *J* = 7.4 Hz, 1H), 7.16 (t, *J* = 7.4 Hz, 1H), 7.14 – 7.02 (m, 3H), 6.97 – 6.84 (m, 3H), 3.73 (s, 3H), 1.38 (s, 6H). ^{13}C NMR (101 MHz, DMSO) δ 155.29, 154.30, 152.89, 145.47, 139.65, 136.60, 129.78, 127.34, 125.89, 122.90, 121.36, 120.86, 119.02, 115.07, 114.19, 109.53, 55.69, 46.63, 27.63. Anal. calcd. for: $C_{22}H_{21}NO$: C, 83.78; H, 6.71; N, 4.44; found: C, 83.94; H, 6.66; N, 4.42.

1,2-bis{3,6-bis[*N*-(9,9-dimethylfluoren-2-yl)-*N*-(4-methoxyfenil)amino]-9*H*-carbazol-9-yl}cyclobutane (V1361)

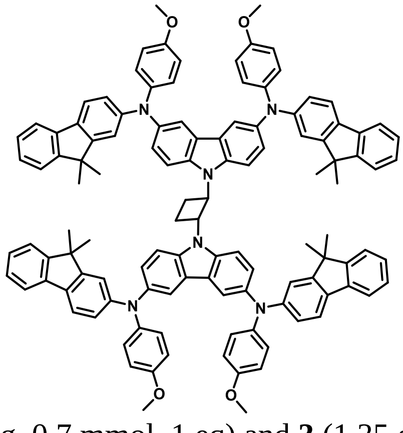

A solution of compound **2** (0.5 g, 0.7 mmol, 1 eq) and **3** (1.35 g, 4.3 mmol, 6 eq) in anhydrous toluene (10 mL) was purged with argon for 30 minutes. Afterwards, palladium (II) acetate (0.02 eq), tri-*tert*-butylphosphonium tetrafluoroborate (0.027 eq) and sodium *tert*-butoxide (6

eq) were added and the solution was refluxed under argon atmosphere for 5 hours. After cooling to room temperature, reaction mixture was filtered through celite, extracted with ethylacetate and distilled water. The organic layer was dried over anhydrous Na_2SO_4, filtered and solvent evaporated. The crude product was purified by column chromatography using 5.5:19.5 v/v THF/*n*-hexane as an eluent. The obtained product was dissolved in THF and precipitated into 15 times excess of *n*-hexane. The precipitate was filtered off and washed with hexane to collect **V1361** as a yellow-green solid. (0.67 g, 57.3%). ^{1}H NMR (400 MHz, DMSO-d_6) δ 7.88 (d, *J* = 8.4 Hz, 4H), 7.78 (s, 4H), 7.55 (d, *J* = 7.8 Hz, 4H), 7.49 (d, *J* = 7.8 Hz, 4H), 7.28 (d, *J* = 7.2 Hz, 4H), 7.24 – 7.16 (m, 8H), 7.11 (t, *J* = 7.4 Hz, 4H), 7.00 (d, *J* = 8.6 Hz, 8H), 6.93 (s, 4H), 6.80 (d, *J* = 8.6 Hz, 8H), 6.69 (d, *J* = 8.4 Hz, 4H), 6.42 – 6.23 (m, 2H), 3.64 (s, 12H), 2.92 – 2.77 (m, 2H), 2.76 – 2.56 (m, 2H), 1.17 (s, 24H). ^{13}C NMR (101 MHz, DMSO) δ 155.83, 154.82, 153.15, 149.03, 141.15, 140.42, 139.09, 137.50, 131.40, 127.37, 126.76, 126.35, 125.32, 123.94, 122.86, 121.12, 119.41, 119.19, 118.17, 115.27, 114.25, 111.87, 55.55, 53.96, 46.56, 27.29, 27.25. Anal. calcd for $C_{116}H_{98}N_6O_2$: C, 84.95; H, 6.02; N, 5.12; found: C, 84.85; H, 6.06; N, 5.15. $C_{116}H_{98}N_6O_2[M^+]$ exact mass = 1638.76, MS (ESI) = 1639.48.

1,2-bis{3,6-bis[*N*-(9-ethylcarbazol-3-yl)-N-(4-methoxyfenil)amino]-9*H*-carbazol-9-yl}cyclobutane (V1366)

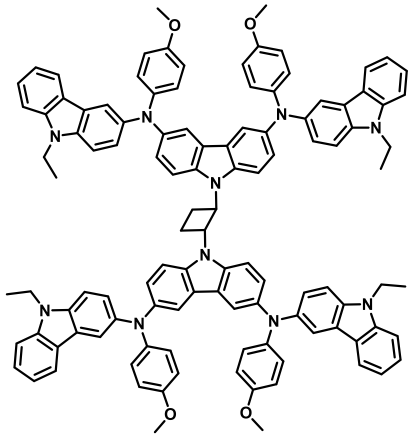

A solution of compound **2** (0.5 g, 0.7 mmol, 1 eq) and 9-ethyl-*N*-(4-methoxyphenyl)-9*H*-carbazol-3-amine[1] (1.35 g, 4.3 mmol, 6 eq) in anhydrous toluene (10 mL) was purged with argon for 30 minutes. Afterwards, palladium (II) acetate (0.02 eq), tri-*tert*-butylphosphonium tetrafluoroborate (0.027 eq) and sodium *tert*-butoxide (6 eq) were added and the solution was refluxed under argon atmosphere for 5 hours. After cooling to room temperature, reaction mixture was filtered through celite, extracted with ethylacetate and distilled water. The organic layer was dried over anhydrous Na_2SO_4, filtered and solvent evaporated. The crude product was purified by column chromatography using 4.5:8 v/v THF/*n*-hexane as an eluent. The obtained product was dissolved in THF and precipitated into 15 times excess of *n*-hexane. The precipitate was filtered off and washed with hexane to collect **V1366** as a yellow-green solid. (0.71 g, 60.7%). ^{1}H NMR (400 MHz, THF-d_6) δ 7.84 (d, *J* = 8.0 Hz, 4H), 7.75 (s, 4H), 7.69 – 7.58 (m, 8H), 7.37 (d, *J* = 8.4 Hz, 4H), 7.33 – 7.25 (m, 8H), 7.19 – 7.11 (m, 8H), 6.97 (t, *J* = 7.4 Hz, 4H), 6.92 (d, *J* = 8.8 Hz, 8H), 6.68 (d, *J* = 8.8 Hz, 8H), 6.38 – 6.26 (m, 2H), 4.31 (q, *J* = 7.0 Hz, 8H), 3.65 (s, 12H), 3.08 – 2.93 (m, 2H), 2.71 – 2.58 (m, 2H), 1.33 (t, *J* = 7.0 Hz, 12H). ^{13}C NMR (101 MHz, THF) δ 154.55, 143.30, 141.94, 141.50, 140.44, 136.86, 136.20, 125.19, 124.98 124.35, 123.79, 123.69, 123.56, 122.77, 120.21, 118.13, 116.09, 115.97, 114.11, 110.49, 108.88, 108.19, 54.74, 54.52, 37.04, 20.57, 13.14. Anal. calcd for $C_{112}H_{94}N_{10}O_4$: C, 81.82; H, 5.76; N, 8.52; found: C, 81.91; H, 5.70; N, 7.50. $C_{112}H_{94}N_{10}O_4[M^+]$ exact mass = 1642.75, MS (ESI) = 1643.47.

Bis(9-ethyl-9*H*-carbazol-3-yl)amine (4)

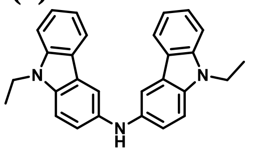

Anhydrous dioxane (14 mL) with few drops of distilled water (0.02 eq) was purged with argon for 20 minutes. After that, the temperature was raised to 80 °C, palladium (II) acetate (0.005 eq) and XPhos (0.015 eq) were added. The mixture was stirred for 1.5 minutes and temperature was raised to 110 °C. 3-bromo-9-ethylcarbazole (2.7 g, 9.8 mmol, 1 eq), 3-amino-9-ethylcarbazole (2.5 g, 11.8 mmol, 1.2 eq) and sodium *tert*-butoxide (1.4 eq) were added and stirred for 30 minutes. After cooling to room temperature, reaction mixture was extracted with ethyl acetate and distilled water. The organic layer was dried over anhydrous Na_2SO_4, filtered and the solvent evaporated. The crude product was purified by column chromatography using 3:22 v/v THF/*n*-hexane as an eluent. Light orange brown crystals were collected as a final product. (3.24 g, 81.6 %). ^{1}H NMR (400 MHz, THF-d_6) δ 7.94 (d, *J* = 7.6 Hz, 2H), 7.81 (s, 2H), 7.42–7.30 (m, 6H), 7.24 (d, *J* = 8.4 Hz, 2H), 7.06 (t, *J* = 7.2 Hz, 2H), 6.89 (s, 1H), 4.37 (q, *J* = 7.2 Hz, 4H), 1.38 (t, *J* = 7.2 Hz, 6H). ^{13}C NMR (101 MHz, THF) δ 140.46, 138.61, 135.27, 124.98, 123.66, 122.90, 119.98, 118.30, 117.79, 108.76, 108.17, 37.04, 13.13. Anal. calcd. for: $C_{28}H_{25}N_3$: C, 83.34; H, 6.24; N, 10.41; found: C, 83.14; H, 6.29; N, 10.49.

1,2-bis{3,6-bis[*N*,*N*-bis(9-ethylcarbazol-3-yl) amino]-9*H*-carbazol-9-yl}cyclobutane (V1367)

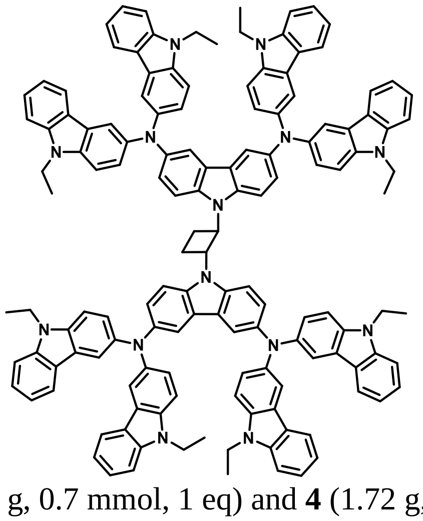

A solution of compound **2** (0.5 g, 0.7 mmol, 1 eq) and **4** (1.72 g, 4.3 mmol, 6 eq) in anhydrous toluene (12 mL) was purged with argon for 30 minutes. Afterwards, palladium (II) acetate (0.02 eq), tri-*tert*-butylphosphonium tetrafluoroborate (0.027 eq) and sodium *tert*-butoxide (6 eq) were added and the solution was refluxed under argon atmosphere for 6 hours. After cooling to room temperature, reaction mixture was filtered through celite and diluted with ethylacetate and distilled water resulting in solid precipitate formed, that was filtered and crude precipitate was purified by column chromatography using 4.5:8 v/v THF/*n*-hexane as an eluent. The obtained product was dissolved in THF and precipitated into 15 times excess of ethanol. The precipitate was filtered off and washed with ethanol to collect **V1367** as a yellow-green solid. (0.62 g, 43.7%). ^{1}H NMR (400 MHz, THF-d_6) δ 7.92 – 7.50 (m, 24H), 7.38 – 7.10 (m, 36H), 6.93 (t, *J* = 7.4 Hz, 8H), 6.46 – 6.29 (m, 2H), 4.24 (q, *J* = 6.8 Hz, 16H), 3.11 – 2.94 (m, 2H), 2.70 – 2.57 (m, 2H), 1.28 (t, *J* = 6.8 Hz, 24H). ^{13}C NMR (101 MHz, THF) δ 142.76, 142.40, 140.41, 136.70, 135.94, 128.72, 127.96, 125.07, 124.46, 123.66, 123.32, 122.85, 120.24, 118.04, 115.74, 115.44, 110.45, 108.83, 108.10, 54.73, 37.01, 13.17. Anal. calcd for $C_{140}H_{114}N_{14}$: C, 84.39; H, 5.77; N, 9.84; found: C, 84.28; H, 5.83; N, 9.89. $C_{140}H_{114}N_{14}[M^+]$ exact mass = 1990.94, MS (ESI) = 1991.12.

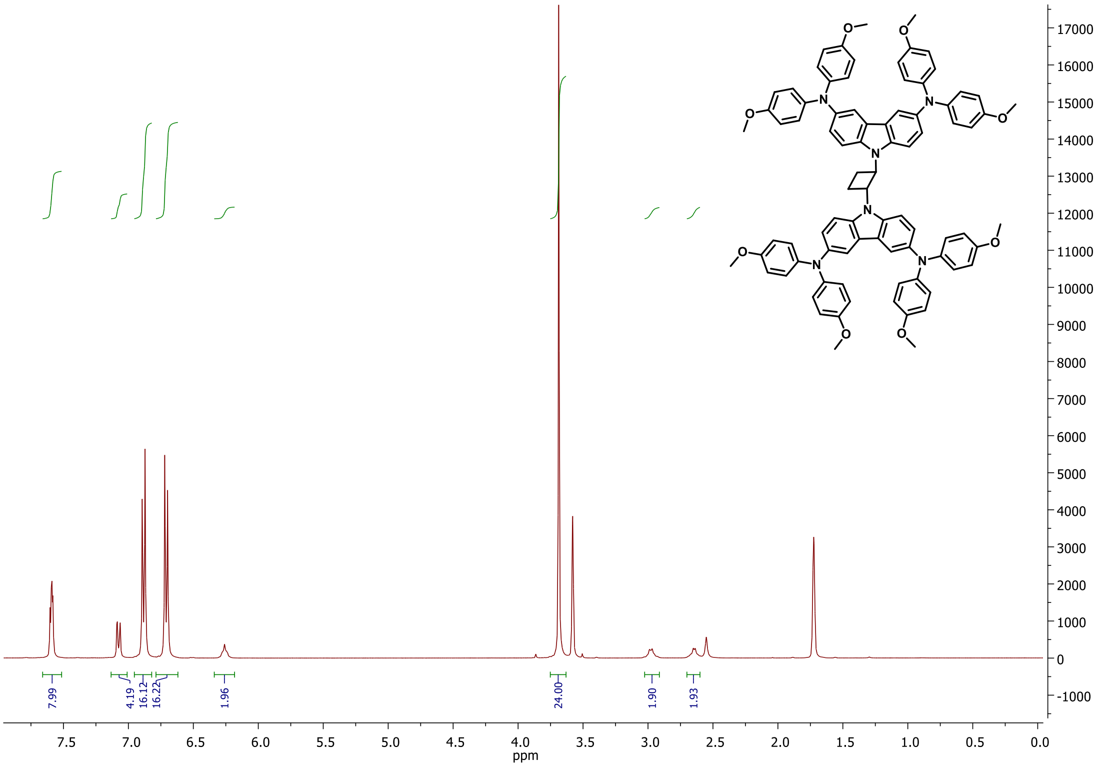
7.99
4.19
16.12
16.22
1.96
24.00
1.90
1.93
ppm
N
O

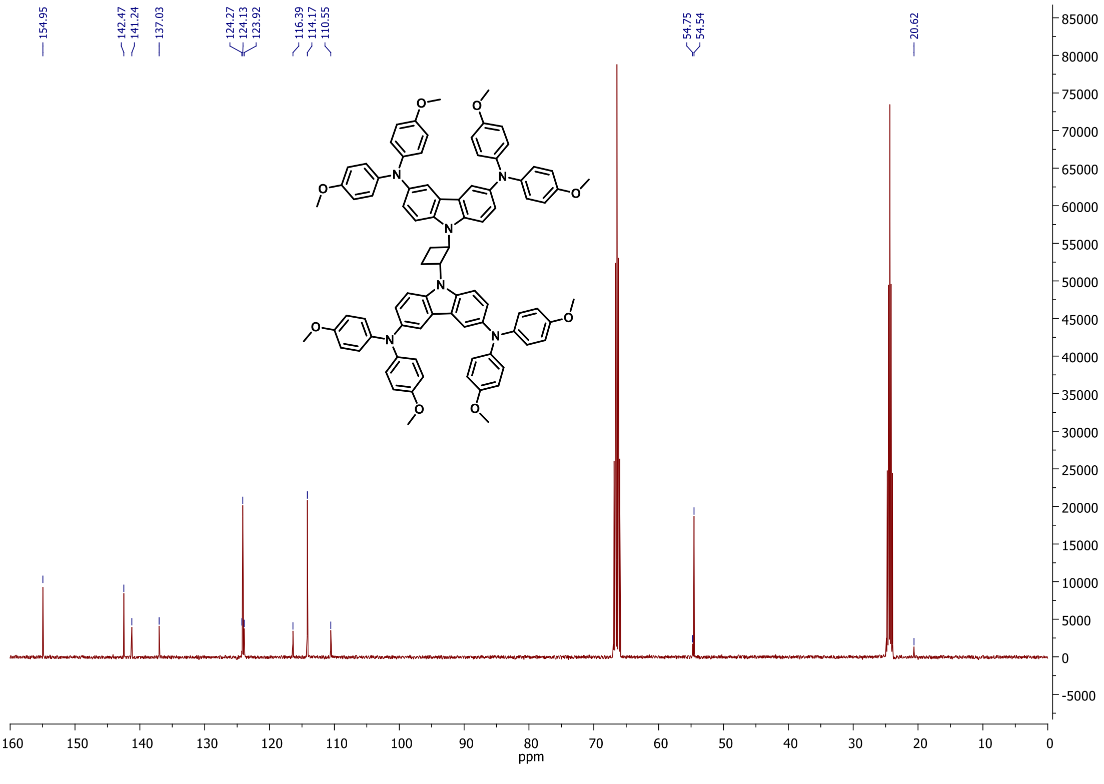
154.95
142.47
141.24
137.03
124.27
124.13
123.92
116.39
114.17
110.55
54.75
54.54
20.62
ppm
N
O

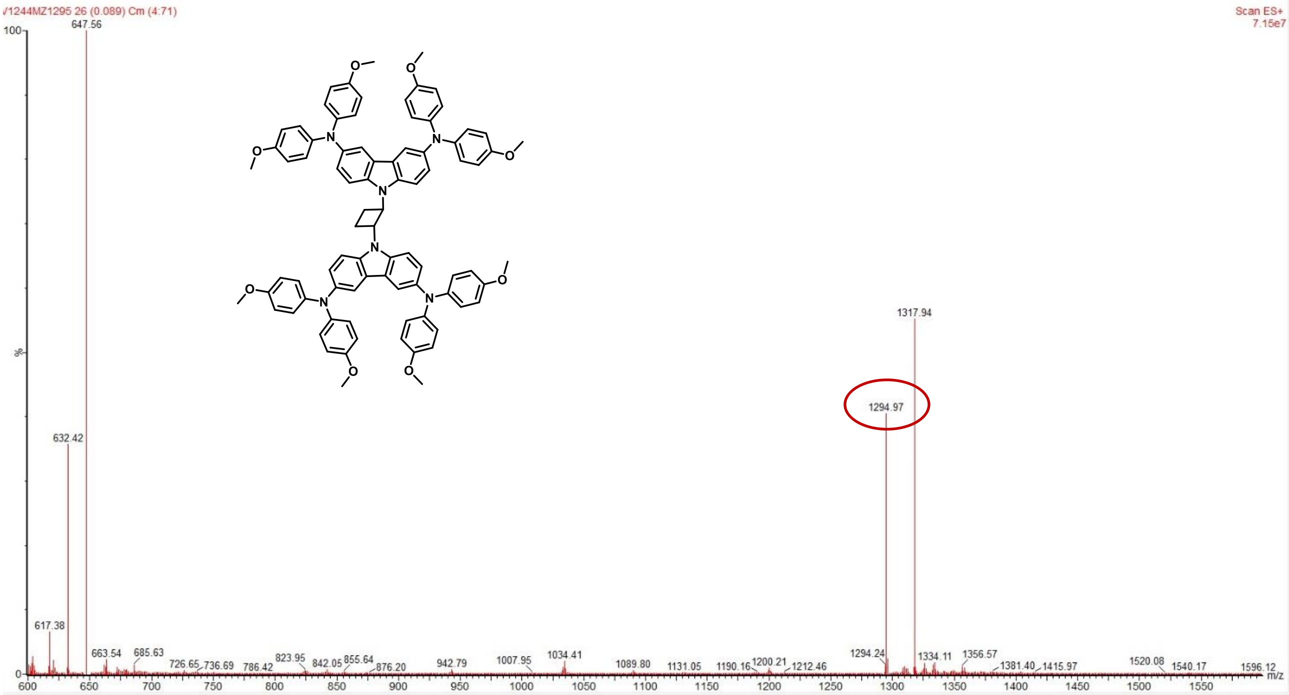

Figure S2. ^{1}H, ^{13}C NMRs and MS spectra of **V1244**.

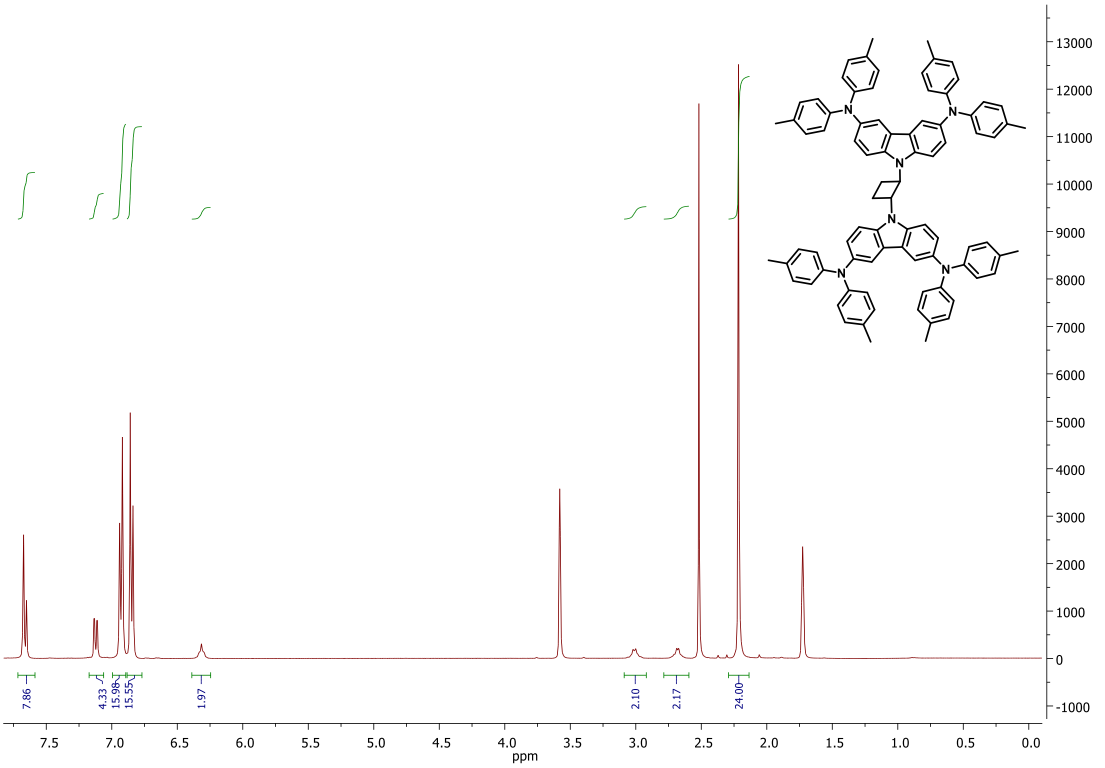

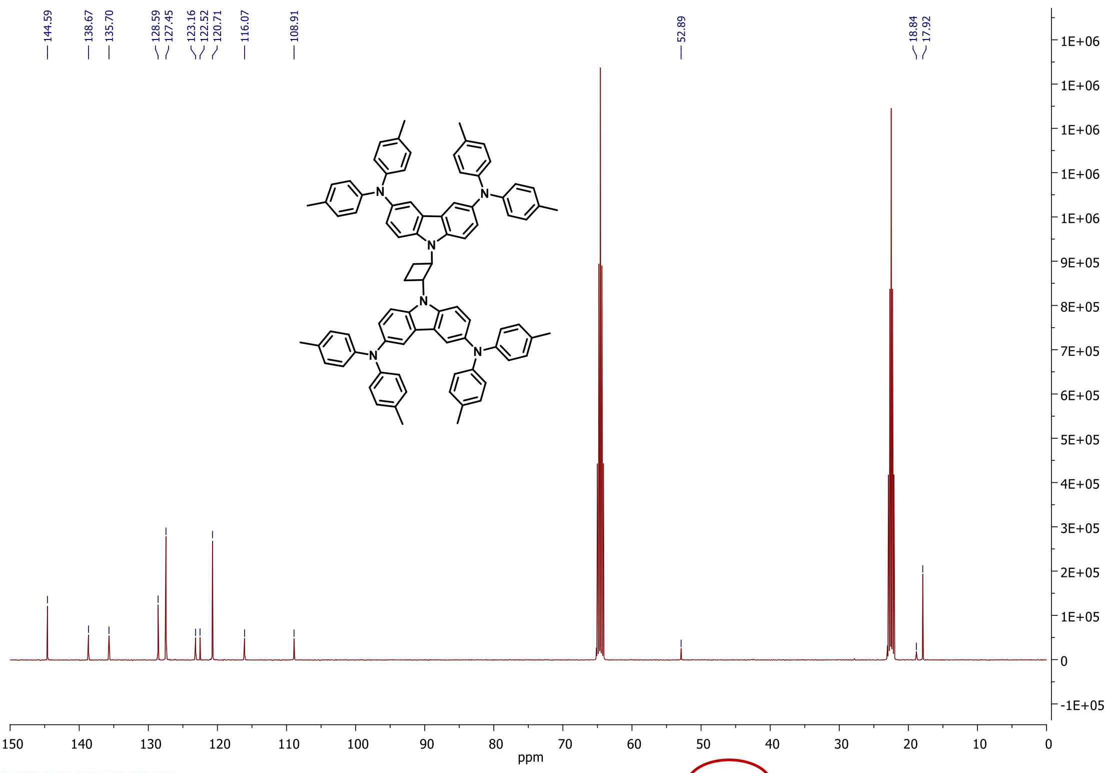

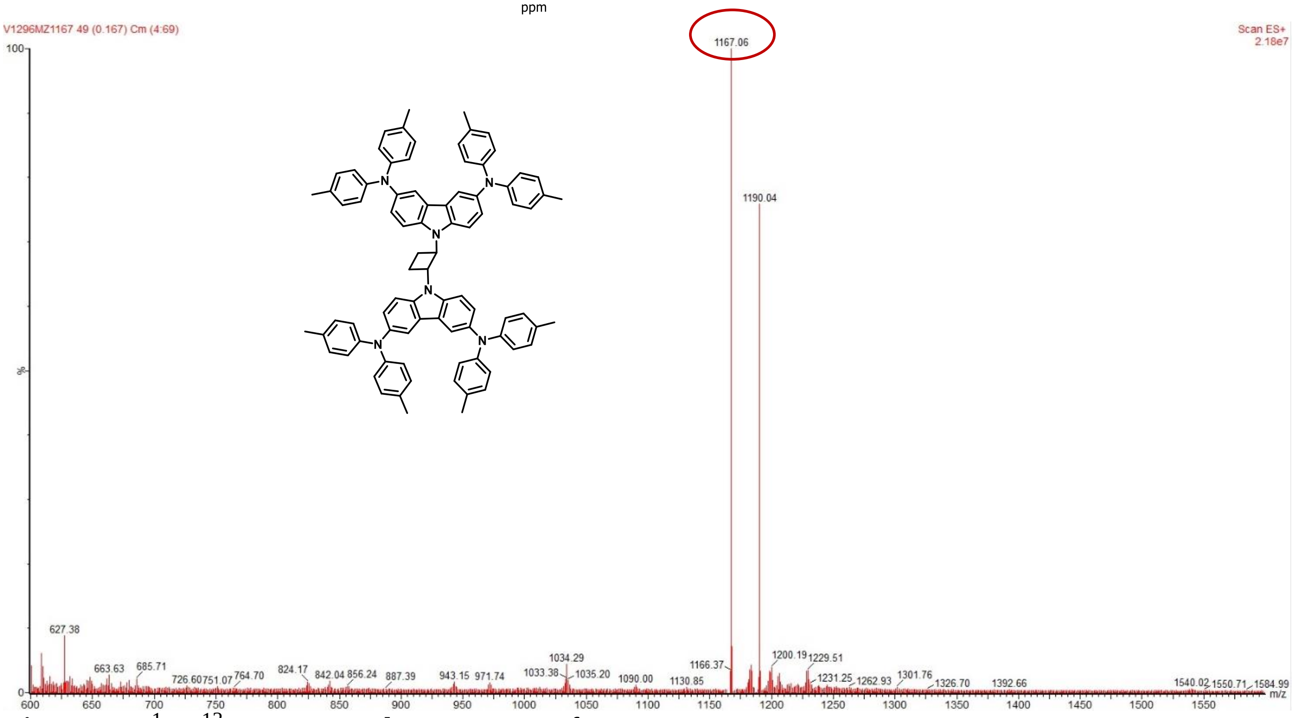

Figure S3. ^{1}H, ^{13}C NMRs and MS spectra of **V1296**.

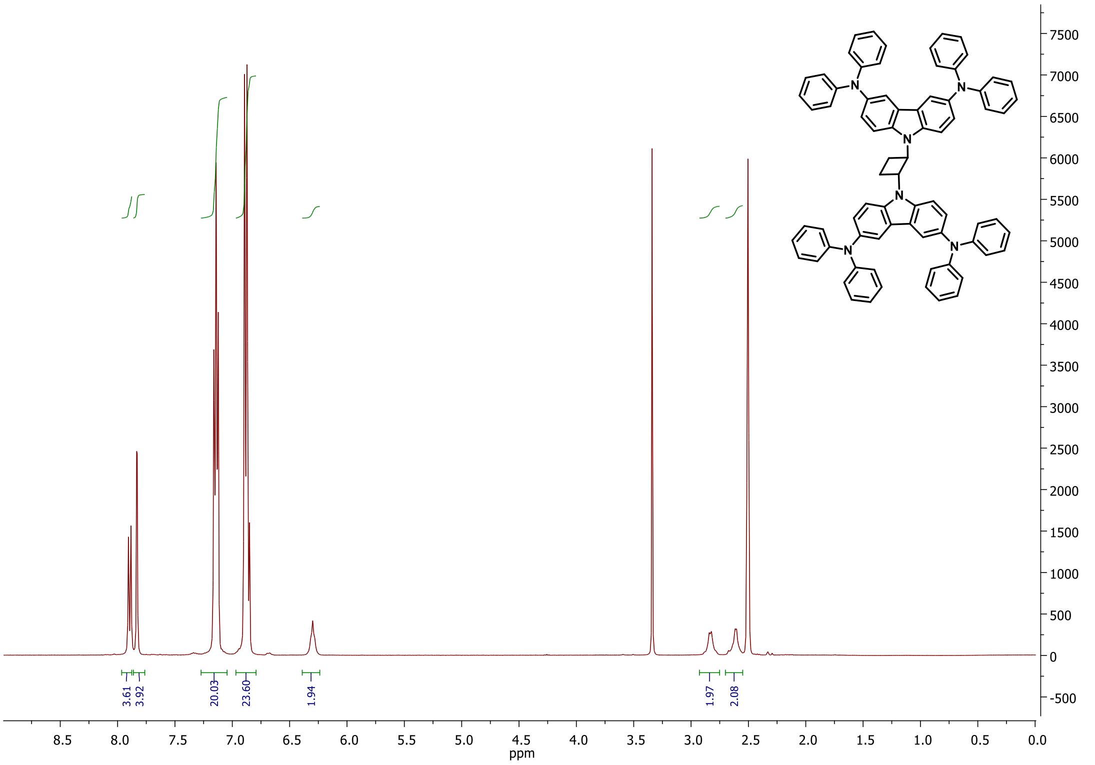
3.61
3.92
20.03
23.60
1.94
1.97
2.08
ppm
N

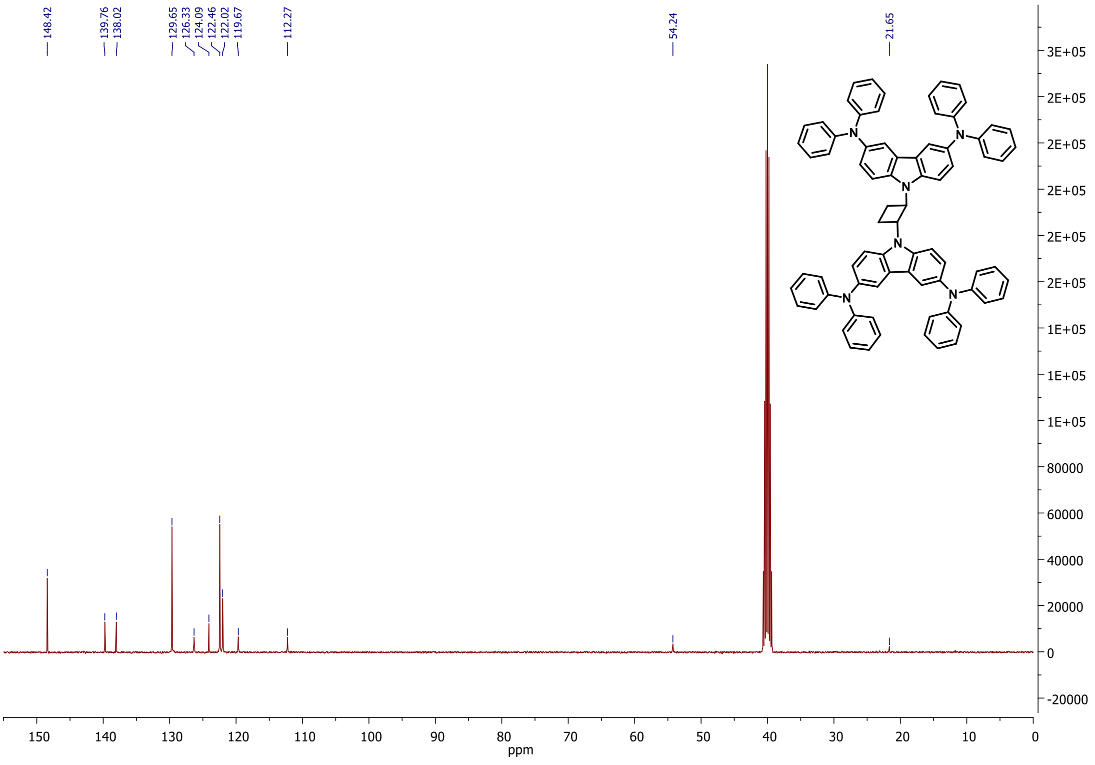
148.42
139.76
138.02
129.65
126.33
124.09
122.46
122.02
119.67
112.27
54.24
21.65
ppm
N

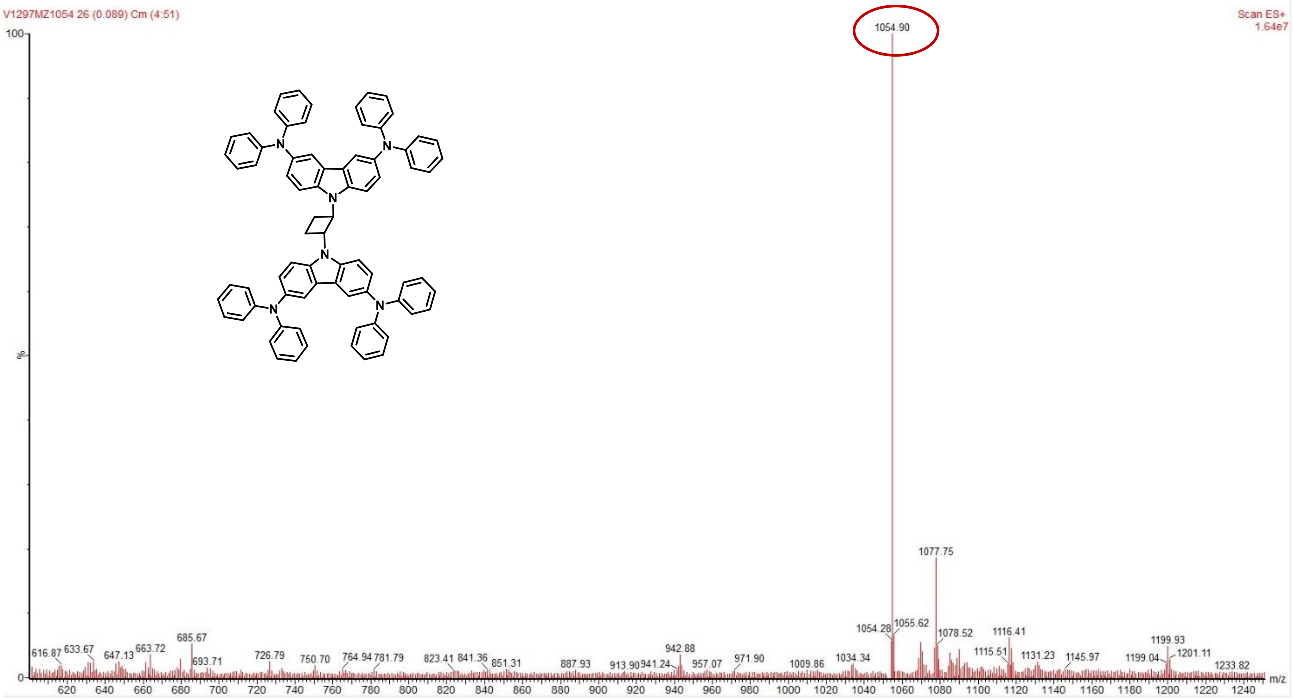

Figure S4. ^{1}H, ^{13}C NMRs and MS spectra of **V1297**.

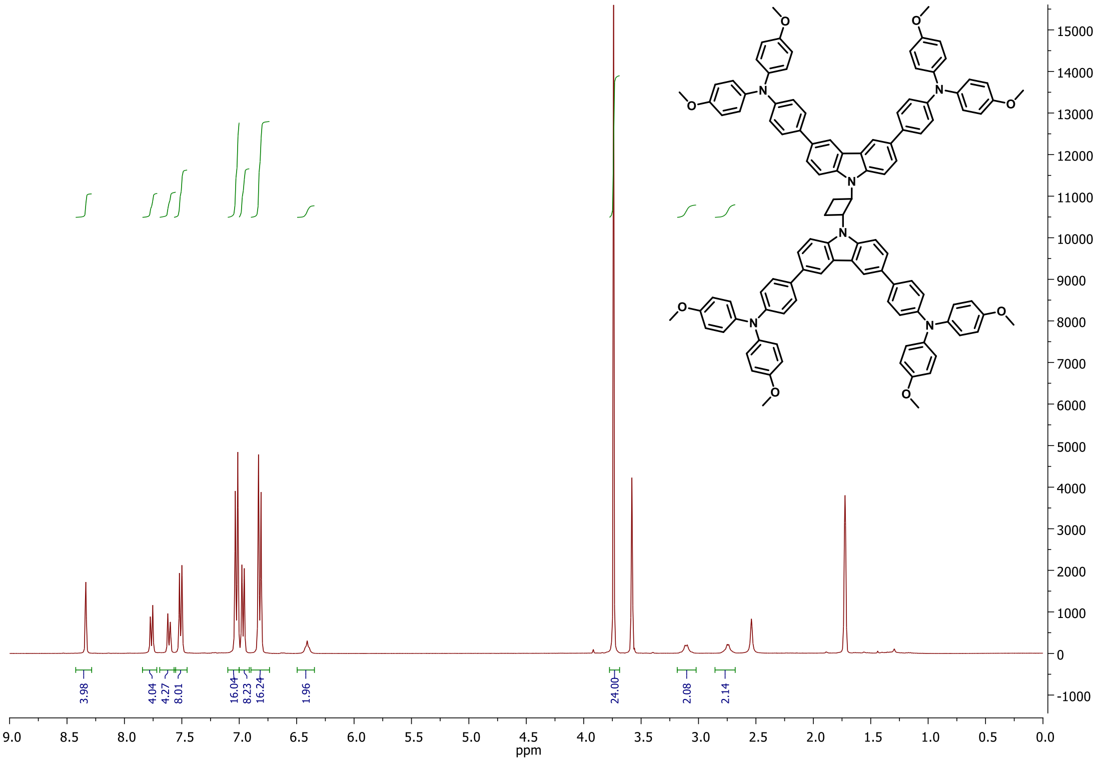

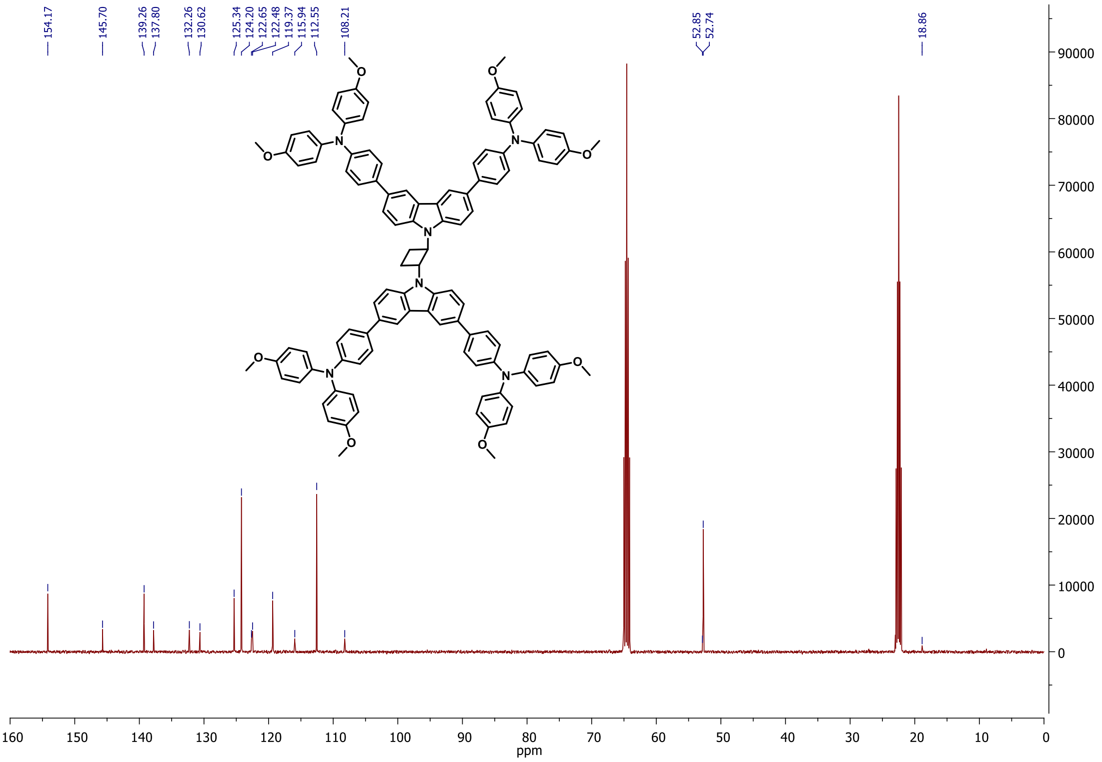

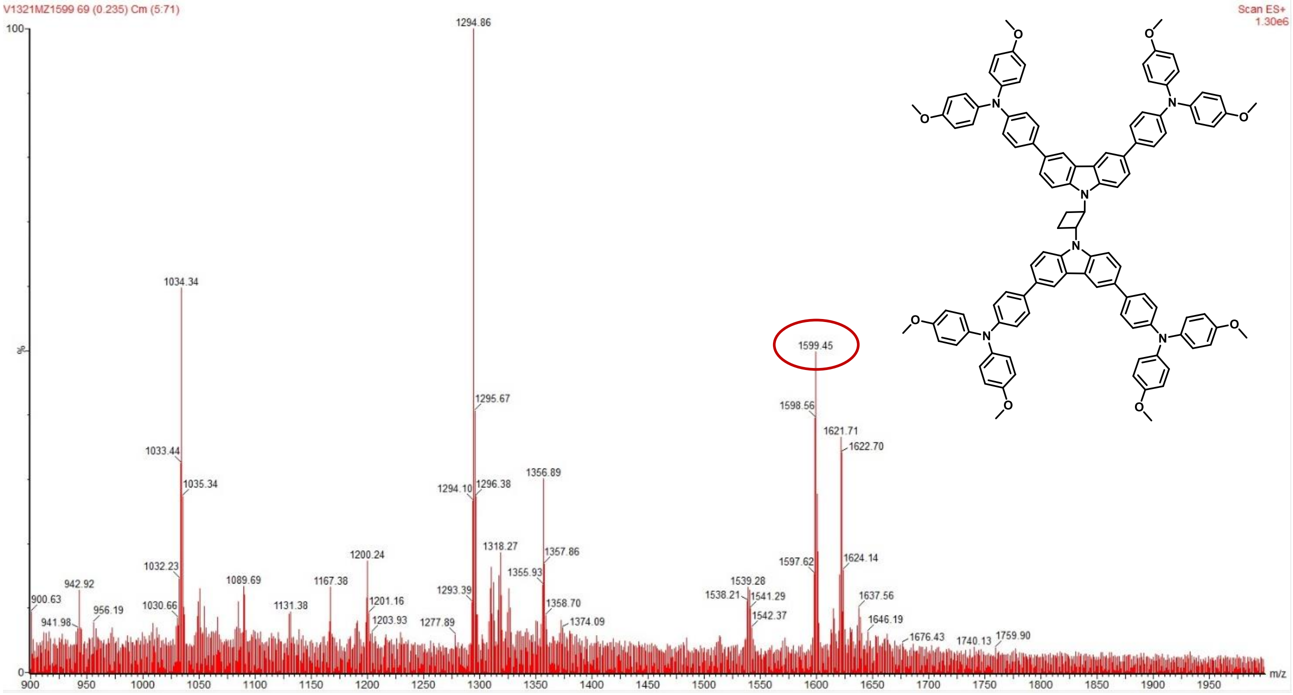

Figure S5. ^{1}H, ^{13}C NMRs and MS spectra of **V1321**.

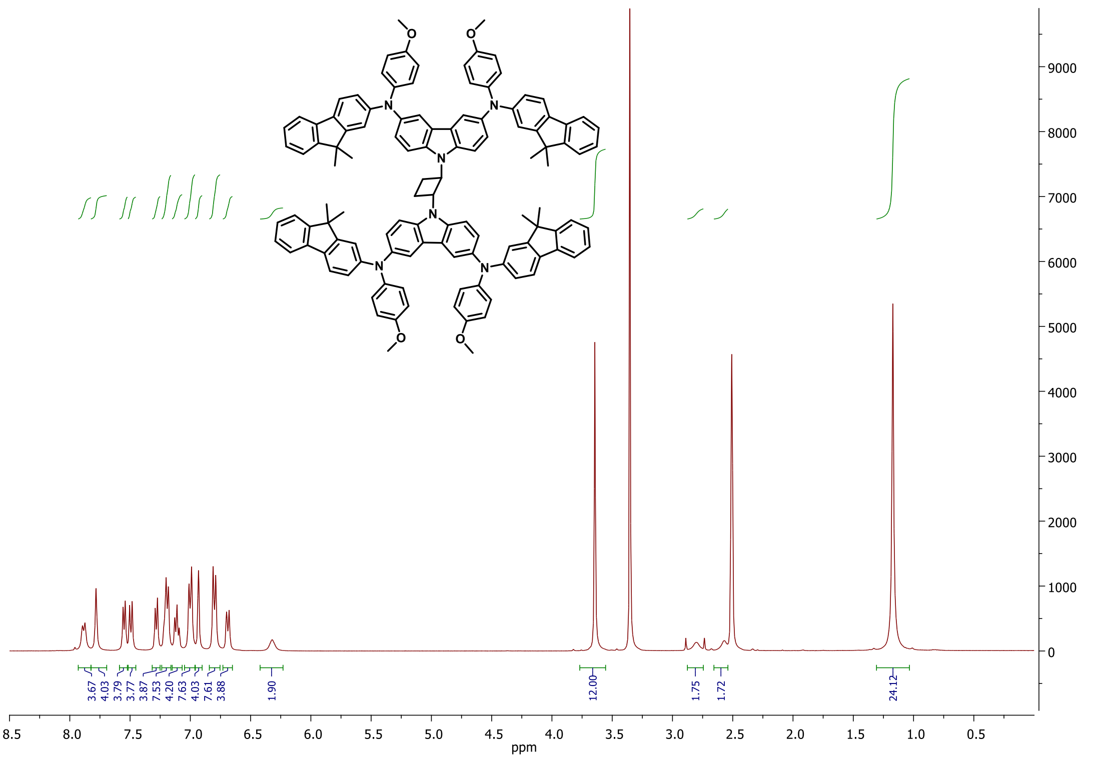
3.67
4.03
3.79
3.77
3.87
7.53
4.20
7.63
4.03
7.61
3.88
1.90
12.00
1.75
1.72
24.12
ppm

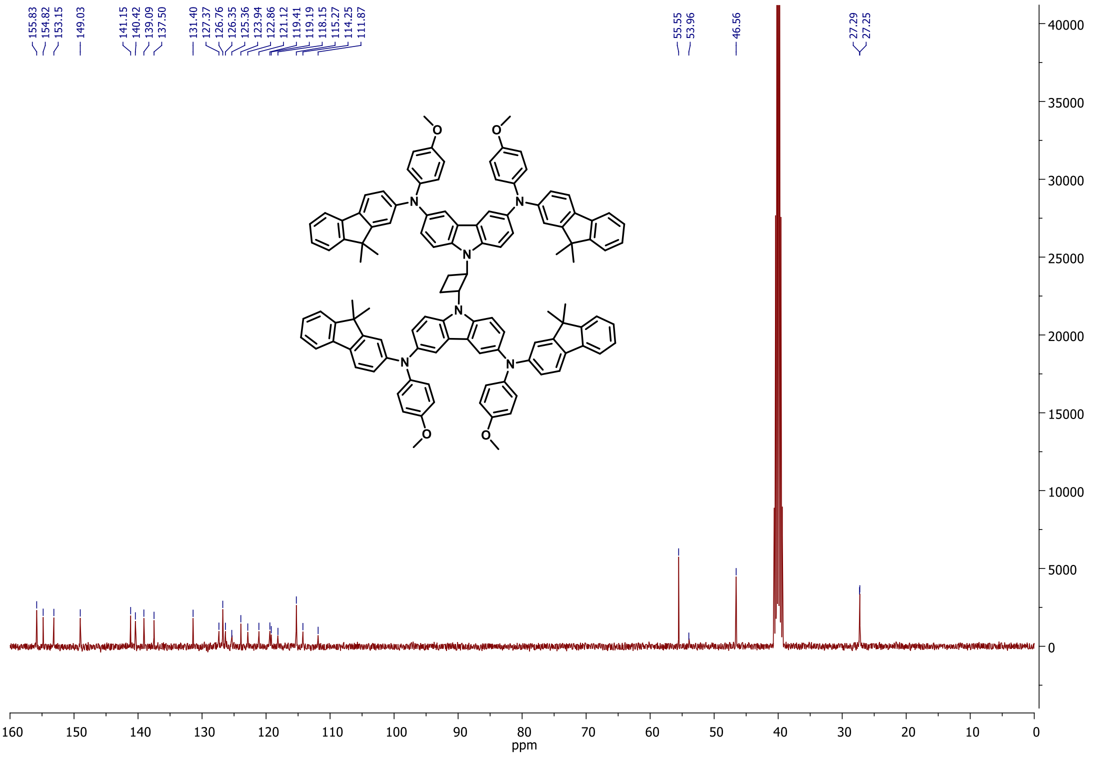
155.83
154.82
153.15
149.03
141.15
140.42
139.09
137.50
131.40
127.37
126.76
126.35
125.36
123.94
122.86
121.12
119.41
119.19
118.15
115.27
114.25
111.87
55.55
53.96
46.56
27.29
27.25
ppm

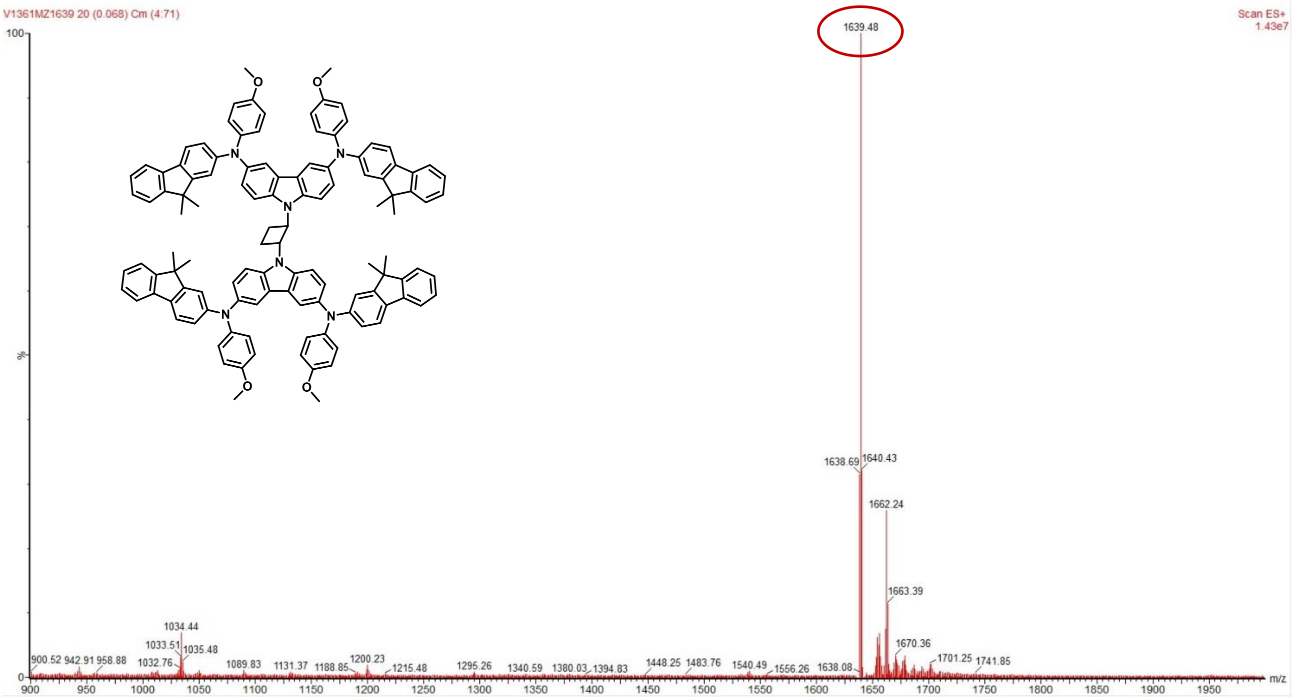

Figure S6. ^{1}H, ^{13}C NMRs and MS spectra of **V1361**

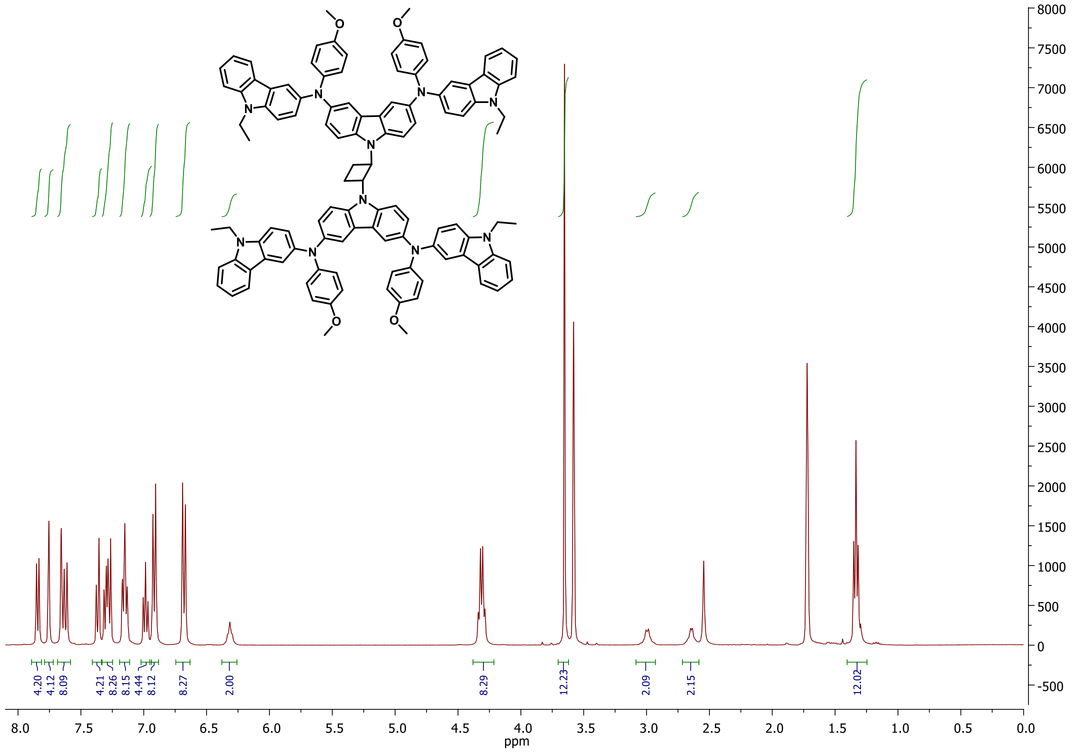

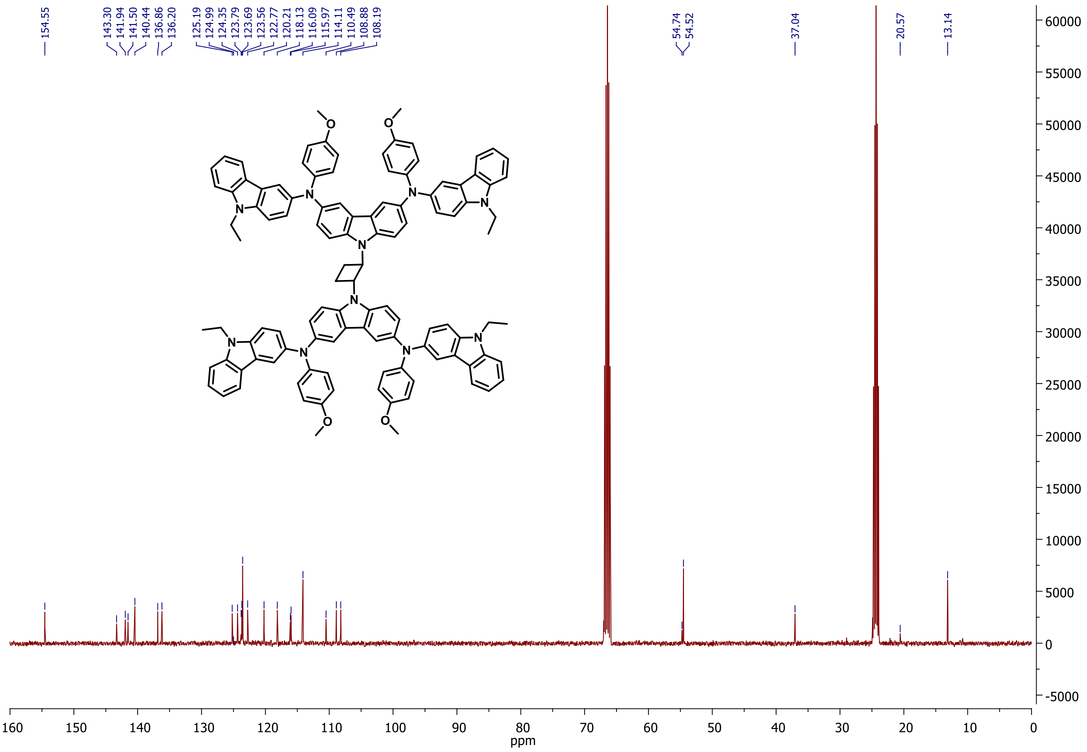

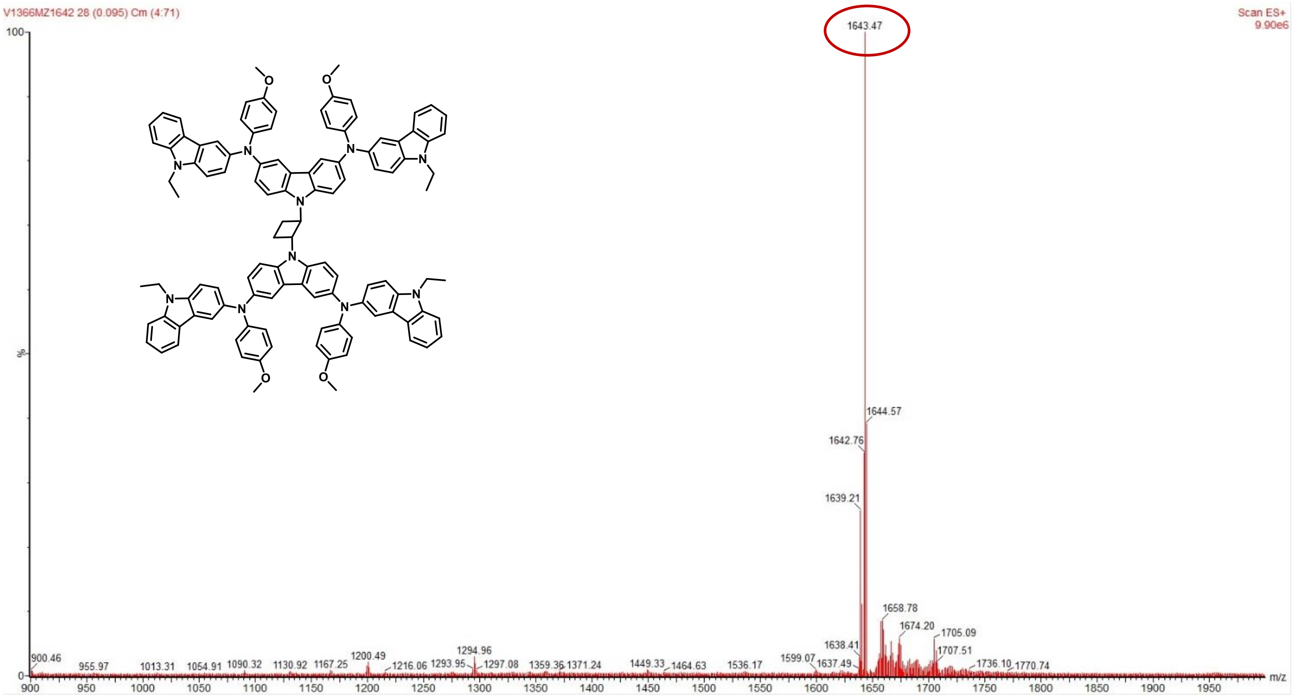

Figure S7. ^{1}H, ^{13}C NMRs and MS spectra of **V1366**.

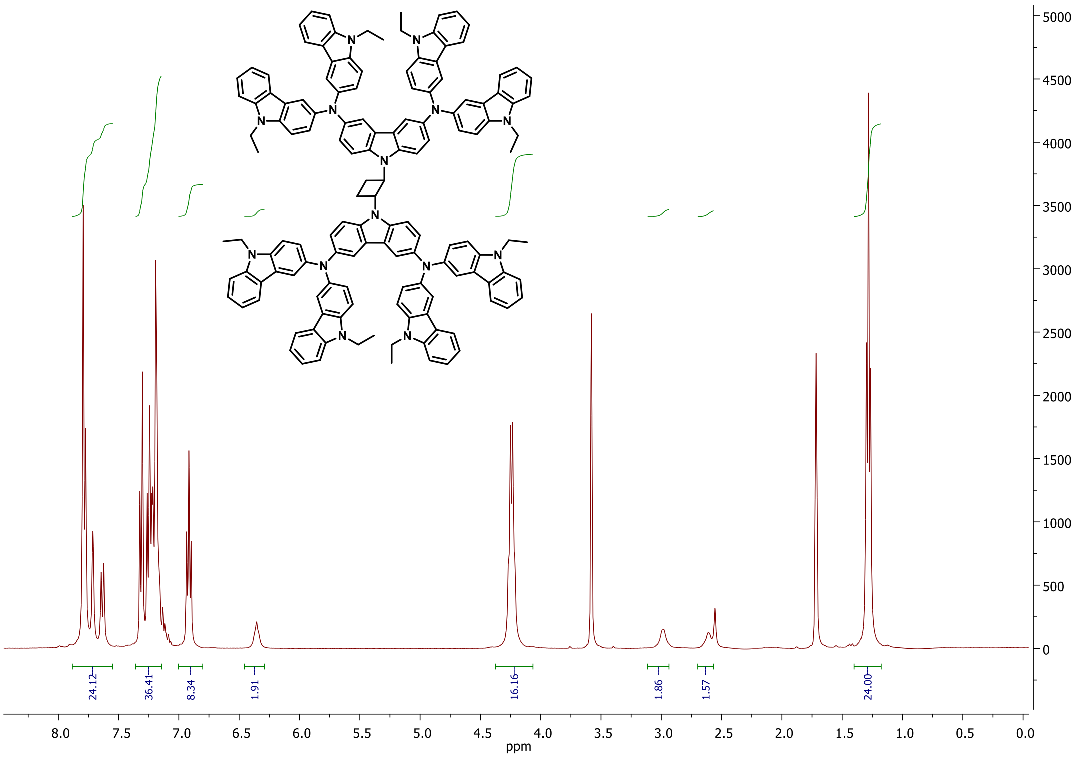
24.12
36.41
8.34
1.91
16.16
1.86
1.57
24.00
ppm
8.0
7.5
7.0
6.5
6.0
5.5
5.0
4.5
4.0
3.5
3.0
2.5
2.0
1.5
1.0
0.5
0.0
5000
4500
4000
3500
3000
2500
2000
1500
1000
500
0

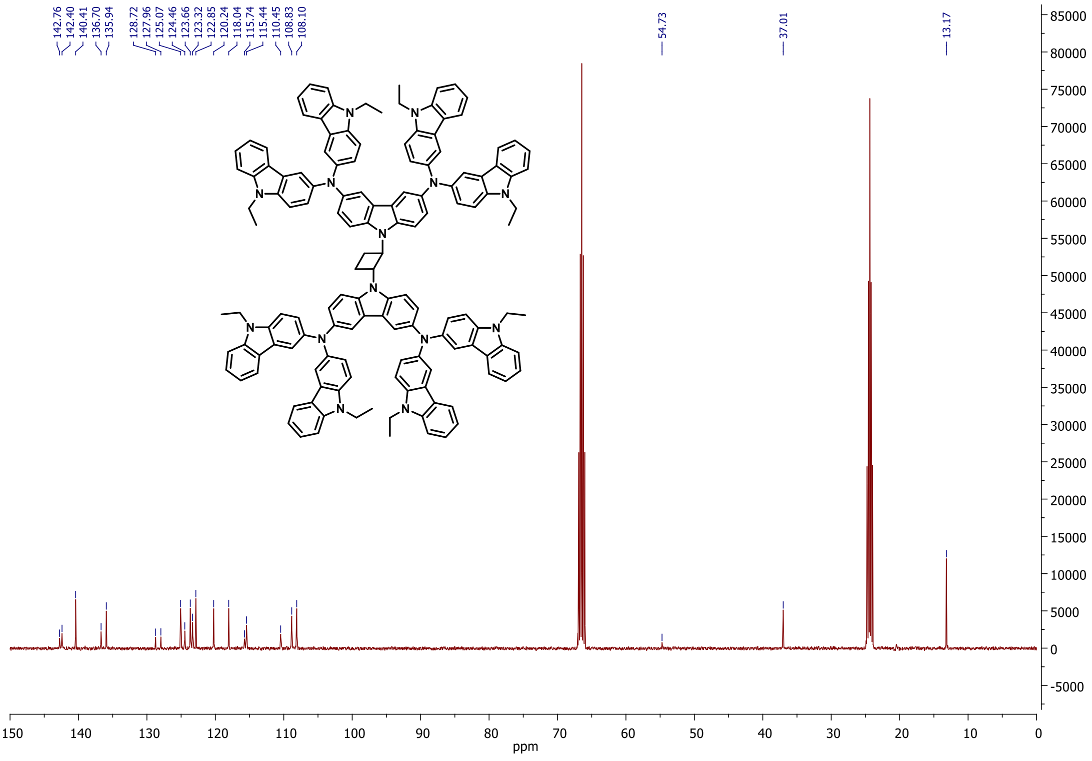
142.76
142.40
140.41
136.70
135.94
128.72
127.96
125.07
124.46
123.66
123.32
122.85
120.24
118.04
115.74
115.44
110.45
108.83
108.10
54.73
37.01
13.17
ppm
150
140
130
120
110
100
90
80
70
60
50
40
30
20
10
0
85000
80000
75000
70000
65000
60000
55000
50000
45000
40000
35000
30000
25000
20000
15000
10000
5000
0
-5000

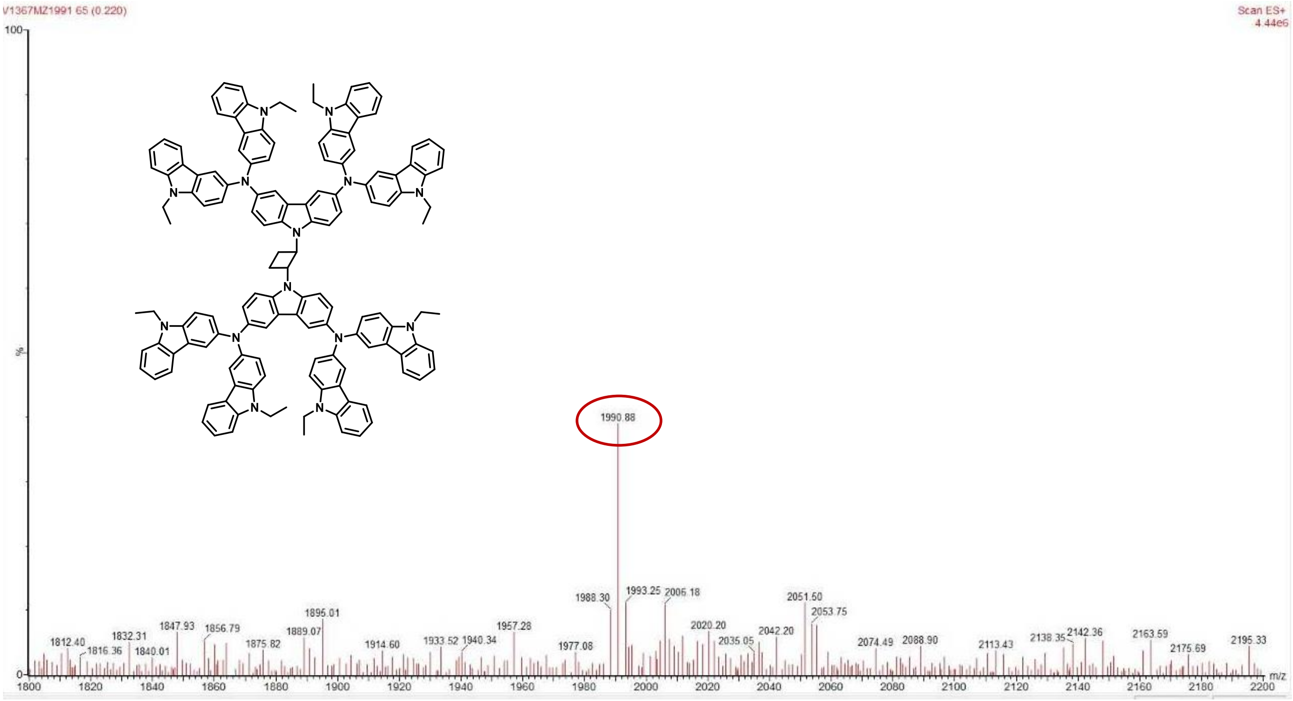

Figure S8. ^{1}H, ^{13}C NMRs and MS spectra of **V1367**.

X-Ray Crystallography

Single crystals of **V1296** were grown by acetone vapor diffusion into saturated chloroform solution at 7 °C. Suitable crystal was selected and mounted on a micromount (MiTeGen) by using inert cryogenic vacuum grease (Apiezon N) as adhesive and analyzed on a XtaLab Synergy diffractometer equipped with HyPix-6000HE hybrid photon counting detector and PhotonJet microfocus X-ray source delivering CuKα (λ = 1.54184) radiation. X-ray diffraction measurements were carried out at 100.0 K using Oxford Cryostream 800 cooling system. Data were collected and processed using CrysAlisPro software. The structure was solved by Intrinsic Phasing with the ShelXT[2] program and refined with the ShelXL[3] package using Least Squares minimization employing Olex2 graphical interface[4]. Due to the presence of highly disordered solvent molecules in crystal lattice solvent masking was applied during the structure refinement. The structure files of **V1296** crystal were deposited with the Cambridge Crystallographic Data Centre (CCDC 2087759) and are available free of charge.

Table S1. Crystallographic data of **V1296** crystals.

Compound	V1296	Z'	0.5
Formula	$C_{84}H_{74}N_6$ + solvent	Wavelength/Å	1.54184
$\rho_{calc.}$/ g cm^{-3}	1.139	Radiation type	Cu K□
μ/mm^{-1}	0.507	Θ_{min}/°	4.263
Formula Weight	1167.49	Θ_{max}/°	67.080
Colour	green	Measured Reflections	21051
Shape	needle	Independent Reflections	6088
Size/mm^3	0.37×0.04×0.02	Reflections with I > 2(I)	4611
T/K	100.00(10)	R_{int}	0.0357
Crystal System	orthorhombic	Parameters	410
Space Group	Pbcn	Restraints	0
a/Å	10.54500(10)	Largest Peak	0.403
b/Å	20.7442(4)	Deepest Hole	-0.254
c/Å	31.1322(4)	Goodness-of-fit	1.029
α/°	90	wR_2 (all data)	0.1741
β/°	90	wR_2	0.1606
γ/°	90	R_1 (all data)	0.0759
V/Å^3	6810.09(17)	R_1	0.0586
Z	4	CCDC number	2087759

Table S2. Fitting parameters for the time-resolved PL spectra.

Equation	y = A1*exp(-x/t1) + A2*exp(-x/t2) + y0		
Sample	Perovskite	Perovskite/V1366	Perovskite/Spiro
y0	0 ± 0	0 ± 0	0 ± 0
A1	0.52226 ± 0.04049	0.57817 ± 0.02288	0.74385 ± 0.01803
t1	163.46916 ± 6.98237	100.39413 ± 2.65503	121.45815 ± 2.63757
A2	0.4584 ± 0.04159	0.39839 ± 0.0239	0.23145 ± 0.01926
t2	389.67908 ± 14.77037	252.13548 ± 6.25012	391.63599 ± 18.04886
Reduced Chi-Sqr	1.21E-04	8.30E-05	1.80E-04
R (COD)	0.99781	0.99813	0.99603

Experimental Section for devices

Materials: 4-tertbutylpyridine (tbp), bis(trifluoromethane) sulfonamide lithium salt and FK209 [tris(2-(1*H*-pyrazol-1-yl)-4-*tert*-butylpyridine)-cobalt(III) tris(bis(trifluoromethylsulfonyl) imide) were purchased from Sigma-Aldrich. Formamidine Iodide and Methylammonium Bromide. Lead iodide and Lead Bromide was purchased from TCI. SnO_2 was purchased from Alfa Aesar. 2,2′,7,7′-tetrakis(*N*,*N*-di-4-methoxyphenylamine)-9,9′-spirobifluorene (spiro-OMeTAD) was purchased from Merck. All of the purchased chemicals were used as received without further purification.

Film and Device Fabrication: Chemically etched FTO glass (Nippon Sheet Glass) was cleaned with detergent solution, acetone, and isopropanol. The substrate was spin coated with a thin layer of SnO_2 nanoparticle film at 3000 rpm for 30 s with a ramp-up of 1500 rpm·s^{-1} from a commercially available in water; the weight ratio of SnO_2 solution to water is 1:3. After spin coating, the substrate was immediately dried on a hotplate at 80 °C, and the substrates were then heated at 190 °C for 30 min. After cooling, 1.5 M $(FAPbI_3)_{0.85}(MAPbBr_3)_{0.15}$ perovskite precursor solution was prepared by mixing of PbI_2, $PbBr_2$, MABr and FAI in DMSO/DMF mixed solvent(1/8). And then, perovskite solutions are successively spin-coated on the substrates at 1000 rpm for 10 s and 5000 rpm for 30 s, respectively. 1 ml of di-ether was dropped in 10 s at 5000 rpm. Perovskite films were annealed at 100 °C for 40 min. The reference solution was prepared by dissolving 91 mg of Spiro-OMeTAD (Merck) with additives in 1 mL of chlorobenzene. As additives, 21 µL of Li-bis(trifluoromethanesulfonyl) imide from the stock solution (520 mg in 1 mL of acetonitrile), 16 µL of FK209 [tris(2-(1*H*-pyrazol-1-yl)-4-*tert*-butylpyridine)-cobalt(III) tris(bis (trifluoromethylsulfonyl)imide) (375 mg in 1 mL of acetonitrile) and 36 µL of 4-tertbutylpyridine were added. The new HTM solutions were prepared by dissolving 40 mM of them with additives in 1 mL of chlorobenzene. As additives, 15 µL of Li-bis(trifluoromethanesulfonyl) imide from the stock solution, 10 µL of FK209 and 26 µL of 4-*tert*-butylpyridine were added. The HTM layers were formed by spin-coating the solution at 4000 rpm for 20 s, and followed by the deposition of the 70 nm thick Au electrode by a thermal evaporation. All the preparative work to deposit perovskite and HTMs was done inside the glove box filled with nitrogen to minimize the influence of moisture.
The module composed of eight-strip cells connected in series was scribed using a YAG laser from Newport. For fabrication of solar modules, 6.5 cm × 7 cm FTO substrates were patterned by a laser with a power of 1500mW and a scribing width of 80 µm. The substrate was spin coated with a thin layer of SnO_2 nanoparticle film at 3000 rpm for 30 s with a ramp-up of 1500 rpm·s^{-1} from a commercially available solution in water; the weight ratio of SnO_2 solution to water is 1:3. After spin coating, the substrate was immediately dried on a hotplate at 80 °C, and the substrates were then heated at 190 °C for 30 min. After cooling, 1 M $(FAPbI_3)_{0.85}(MAPbBr_3)_{0.15}$ perovskite precursor solution was prepared by mixing of PbI_2, $PbBr_2$, MABr and FAI in DMSO/DMF mixed solvent(1/4). And then, perovskite solutions are successively spin-coated on the substrates at 1000 rpm for 10 s and 4000 rpm for 30 s, respectively. 600 µL of chlorobenzene was dropped in 10 s at 4000 rpm. Perovskite films were annealed at 100 °C for 40 min. The HTM solution were prepared by dissolving 40 mM of V1366 with additives in 1 mL of chlorobenzene. As additives, 15 µL of Li-bis(trifluoromethanesulfonyl) imide from the stock solution, 10 µL of FK209 and 26 µL of 4-tertbutylpyridine were added. The HTM layer was formed by spin-coating the solution at 4000 rpm for 30 s, and followed by the deposition of the 70 nm thick Au electrode by a thermal evaporation. Next, SnO_2/Perovskite/HTM layers were scribed by a laser with a power of 1000 mW and a scribing width of 500 µm. Finally, a gold electrode was deposited by

thermal evaporation, and gold layers were scribed by a laser with a power of 1000 mW and a scribing width of 100 μm.

Characterization: XRD was performed using a D8 Advance diffractometer (Bruker AXS) model in an angle range of $2\theta = 10^{o}$-15° (Bruker Corporation, Billerica, MA, USA). The SEM of film morphology was investigated by using a high-resolution scanning electron microscope (Merlin, Zeiss) equipped with a GEMINI II column and a Schottky Field Emission gun. Images were acquired with an In-Lens Secondary Electron Detector. *Photoluminescence (PL) Measurements:* CW was performed with a spectrophotometer (*Gilden Photonics*) using the lamp or a pulsed source at 460 nm (Ps diode lasers BDS-SM, pulse with < 100 ps, from Photonic Solutions, 20 MHz repetition rate, approx. 500 μm spot radius), respectively. The signal was recorded by a photomultiplier tube. For the PL lifetime measurements, samples are excited with a 408-nm pulsed laser (MDL 300, PicoQuant) with 40 $um \cdot cm^{-2}$ pulse energy density (pulse width 180 ps).

Device Measurement: Current–voltage characteristics were recorded by applying an external potential bias to the cell while recording the generated photocurrent with a digital source meter (Keithley Model 2400). The light source was a 450 W xenon lamp (Oriel) equipped with a SchottK113 Tempax sunlight filter (Praezisions Glas & Optik GmbH) to match the emission spectrum of the lamp to the AM1.5G standard. Before each measurement, the exact light intensity was determined using a calibrated Si reference diode equipped with an infrared cutoff filter (KG-3, Schott). The voltage scan rate was 100 $mV \cdot s^{-1}$ and no device preconditioning such as light soaking or forward voltage bias applied for long time, was applied before starting the measurement. The cells were masked with the active area of 0.891 cm^2 to fix the active area and reduce the influence of the scattered light for the small device. And, for the module, the active area is counted by using Nano Measurer 1.2. IPCE spectra were recorded as functions of wavelength under a constant white light bias of ≈10 $mW \cdot cm^{-2}$ supplied by an array of white light emitting diodes. The excitation beam coming from a 300 W xenon lamp (ILC Technology) was focused through a Gemini-180 double monochromator (Jobin Yvon Ltd) and chopped at ≈2 Hz. The signal was recorded using a Model SR830 DSP Lock-In Amplifier (Stanford Research Systems). All measurements were characterized at

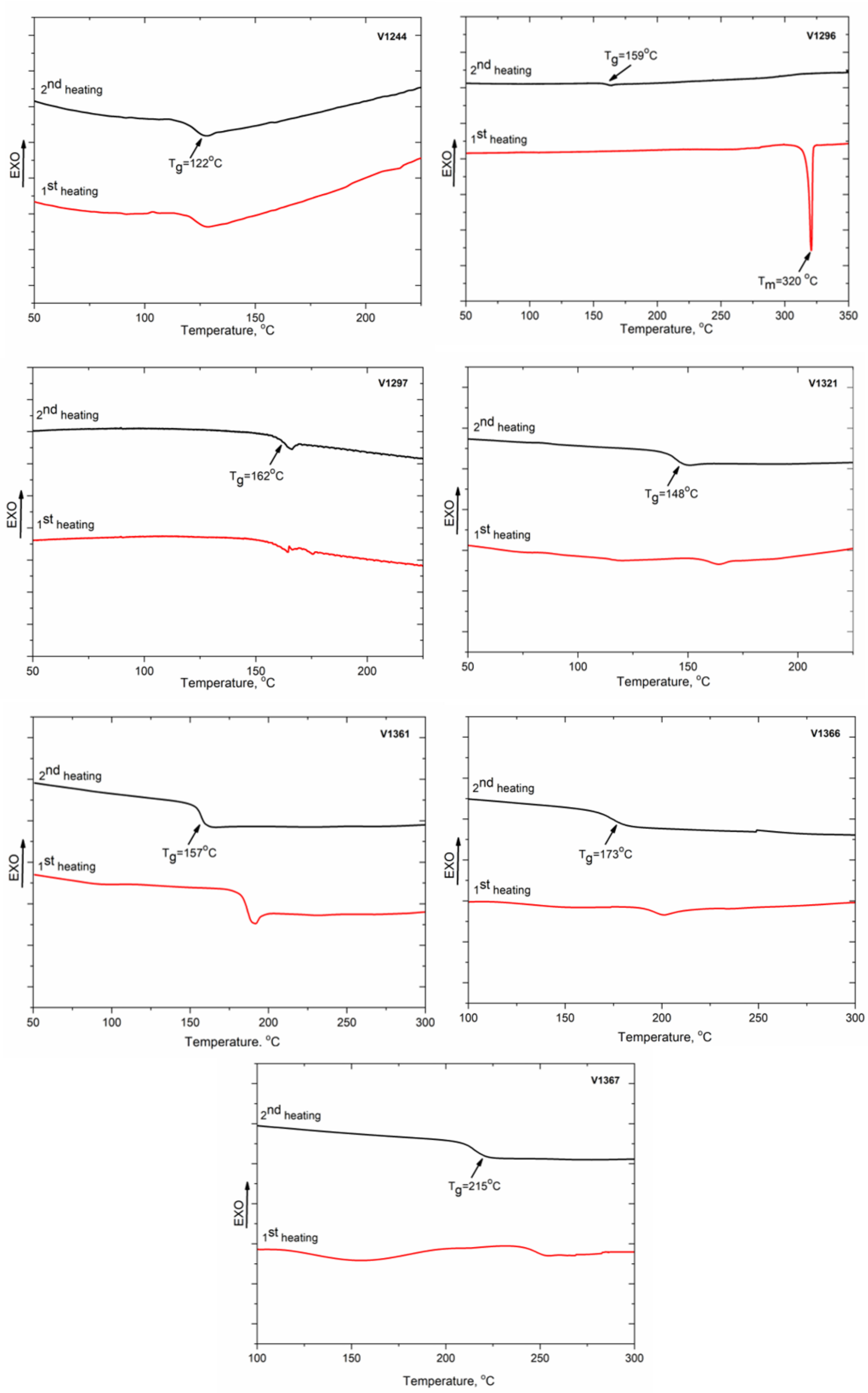

Figure S9. Differential scanning calorimetry (DSC) first and second heating curves of HTMs (scan rate 10 ºC/min, N_2 atmosphere).

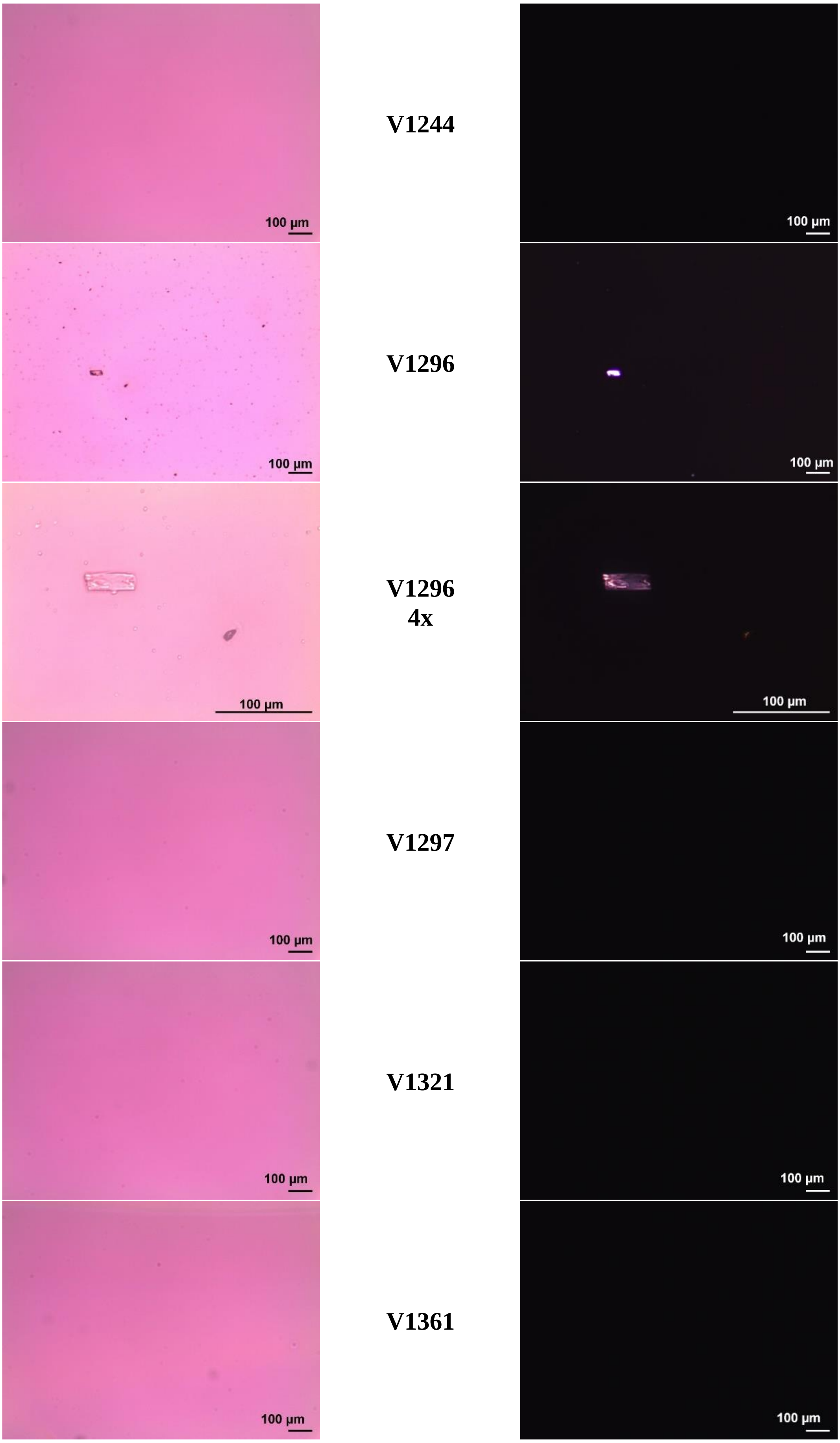
V1244
V1296
V1296
4x
V1297
V1321
V1361
100 µm

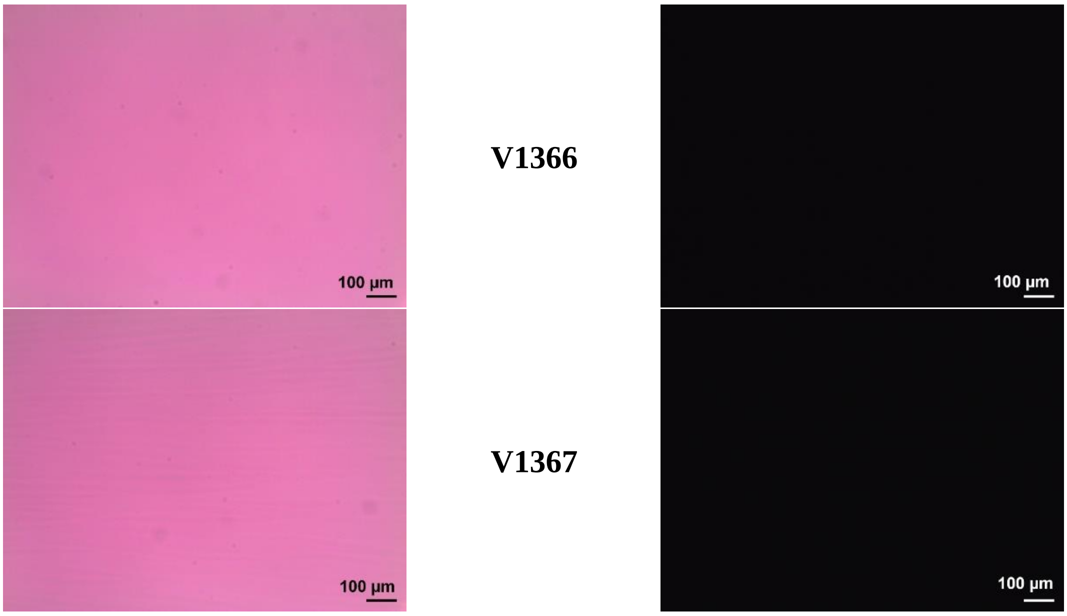

Figure S10. Microscope photographs of the HTM layers; (left) optical micrograph of the film (right) optical micrograph with dark field crossed polarizer.

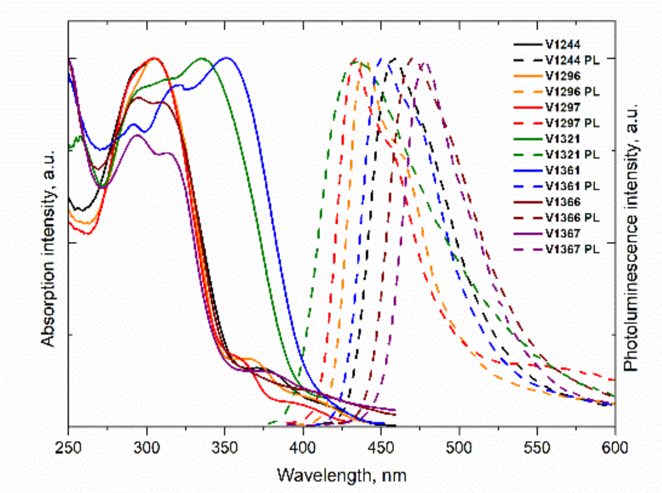

Figure S11. UV−Vis absorption (solid line) and photoluminescence (dashed line) spectra of thin films V-series HTMs.

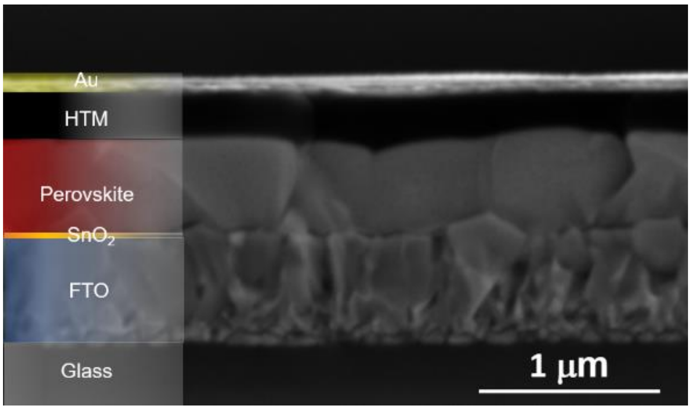

Figure S12. Cross-sectional SEM image of the sample comprising FTO/SnO_2/perovskite/ spiro-OMeTAD/Au layers.

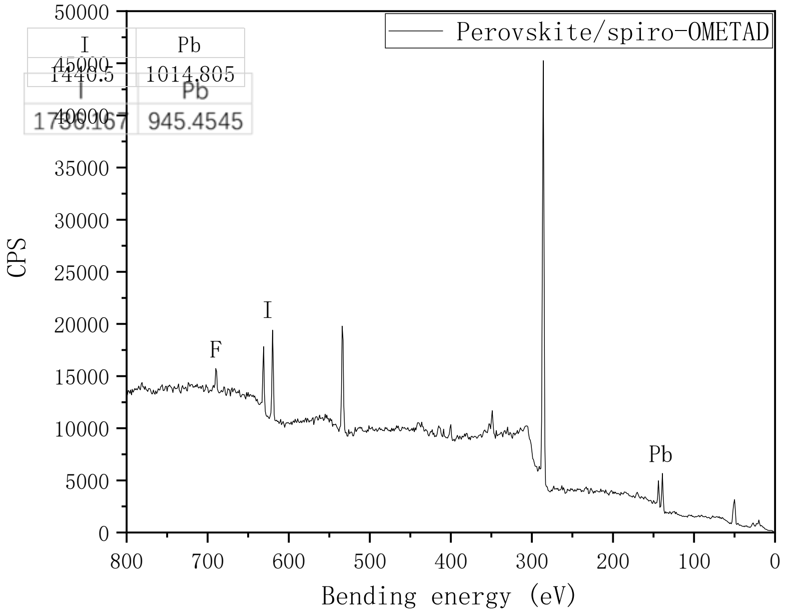

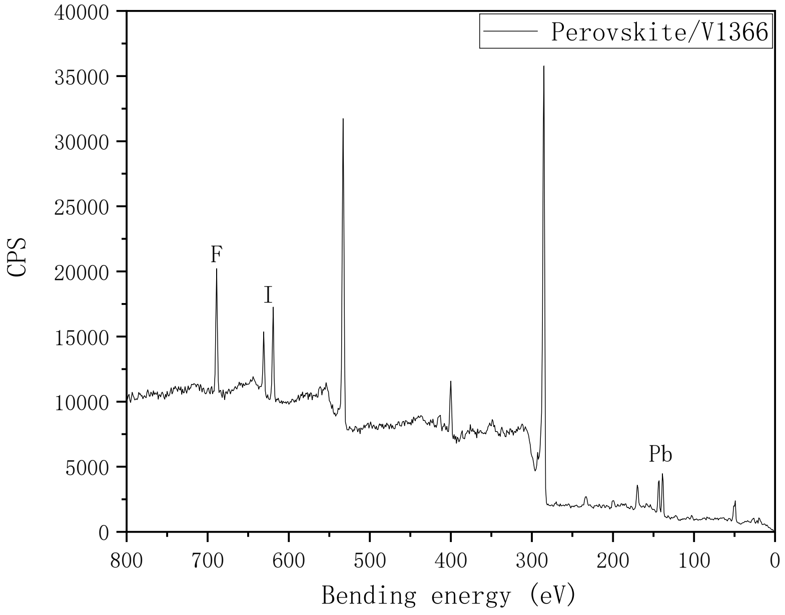

Figure S13. XPS spectra of perovskite/spiro-OMeTAD layer (above) and perovskite/**V1366** layer (below).

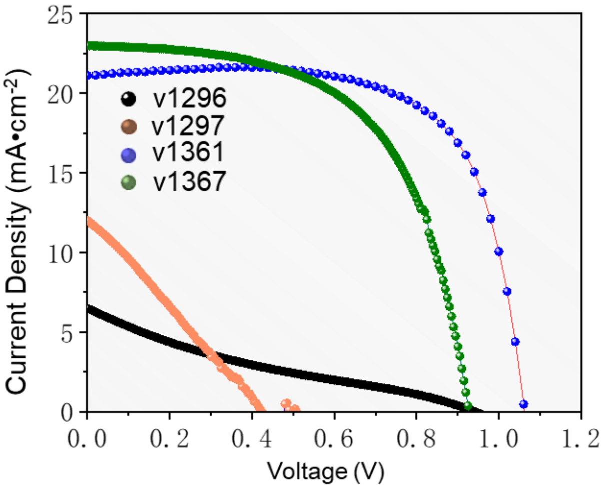

	J_{SC} (mA•cm^{-2})	V_{OC} (V)	FF	PCE (%)
●	6.49	0.942	0.198	1.21
●	12.00	0.506	0.217	1.32
●	21.11	1.061	0.693	15.26
●	22.99	0.927	0.584	12.44

Figure S14. *J*-*V* curves (reverse-scan) of the PSCs based on **V1296**, **V1297**, **V1361** and **V1367** as the HTMs.

Table S3. Photovoltaic parameter of short-circuit photocurrent density (J_{sc}), open-circuit voltage (V_{oc}), fill factor (*FF*) and power conversion efficiency (*PCE*) of 10 samples with **V1366** or spiro-OMeTAD. Temperature and relative humidity were about 20 ~ 30 ºC and 15 ~ 20%.

**V1366**	J_{sc} (mA/cm^2)	V_{oc} (V)	*FF*	*PCE* (%)	Spiro-OMeTAD	J_{sc} (mA/cm^2)	V_{oc} (V)	*FF*	*PCE* (%)
	24.38	1.092	0.791	21		24.17	1.114	0.803	21.64
	24.51	1.059	0.798	20.59		24.41	1.122	0.789	21.48
	24.47	1.08	0.792	20.76		24.32	1.108	0.788	21.11
	24.57	1.082	0.773	20.52		24.22	1.108	0.804	21.57
	24.61	1.098	0.759	20.48		24.21	1.112	0.806	21.7
	24.63	1.099	0.774	20.92		24.23	1.122	0.792	21.49
	24.57	1.082	0.773	20.52		24.1	1.105	0.782	20.79
	24.41	1.092	0.787	20.87		24.21	1.097	0.805	21.35
	24.4	1.087	0.797	21.08		24.25	1.113	0.8	21.55
	24.37	1.09	0.794	21		24.1	1.099	0.801	21.21
	24.38	1.092	0.791	21		24.17	1.114	0.803	21.64
	24.51	1.059	0.798	20.59		24.41	1.122	0.789	21.48
	24.47	1.08	0.792	20.76		24.32	1.108	0.788	21.11
	24.57	1.082	0.773	20.52		24.22	1.108	0.804	21.57

Table S4. Photovoltaic performance of perovskite solar modules with an active area of 10-100 cm^2 and PCE over 15%.

Architecture	Module structure	Active area (cm^2)	PCE (%)	Reference
p-i-n	FTO/NiO/$(CH(NH_2)_2)_{0.85}(CH_3NH_3)_{0.15}Pb(I_{0.85}Br_{0.15})_3$/PCBM/BCP/Ag	33.2 (assumed geometric fill factor 0.92)	16.95	[5]
n-i-p	FTO/SnO_2/$K_xCs_{0.05}(FA_{0.85}MA_{0.15})_{0.95}Pb(I_{0.85}Br_{0.15})_3$/spiro-OMeTAD/Au	20	15.76	[6]
n-i-p	FTO/TiO_2/$CH_3NH_3PbI_3$/spiro-OMeTAD/Au	16.2 (assumed geometric fill factor 0.92)	17.2	[7]
n-i-p	FTO/TiO_2/$(FAPbI_3)_{0.95}(MAPbBr_3)_{0.05}$/P3HT/Au	24.97	16	[8]
n-i-p	FTO/c-TiO_2/m-TiO_2/$MAPbI_3$(Cl)/spiro-OMeTAD/Au	12	15.3	[9]
n-i-p	FTO/TiO_2/$CH_3NH_3PbI_{3-x}Cl_x$/PTAA/Au	40	15.5	[10]
n-i-p	FTO/TiO_2-SnO_2/PCBM/$MAPbI_3$/spiro-OMeTAD/Au	21	18.13	[11]
n-i-p	FTO/c-TiO_2/m-TiO_2/CsMAFA/PTAA/Au	70	15.3	[12]
n-i-p	FTO/SnO_2/$(CsPbI_3)_{0.05}((FAPbI_3)_{0.85}(MAPbBr_3)_{0.15})_{0.95}$/spiro-OMeTAD/Au	25	15.3	[13]
p-i-n	ITO/PTAA/$MAPbI_3$/C_{60}/BCP/Cu	30.8	16.38	[14]
p-i-n	ITO/PTAA/$MAPbI_3$/C_{60}/BCP/metal	58.6 (assumed geometric fill factor 0.92)	17.82	[15]
p-i-n	ITO/PEDOT:PSS/$MAPbI_3$/$PC_{71}BM$/Ca/Al	11.25	15.4	[16]
n-i-p	FTO/c-TiO_2/m-TiO_2/$MAPbI_3$/MoS_2/spiro-OMeTAD/Au	82	15.3	[17]
n-i-p	ITO/SnO_2/$Cs_{0.05}(FA_{0.85}MA_{0.15})_{0.95}Pb(I_{0.85}Br_{0.15})_3$/spiro-OMeTAD/Au	14.78 (assumed geometric fill factor 0.92)	16.54	[18]
p-i-n	PEN/PEDOT:PSS/NiO_x//$PC_{61}BM$/Ag	15	16.15	[19]
n-i-p	ITO/SnO_2/Zn_2SnO_4-SnO_2/$(FAPbI_3)_{0.95}(MAPbBr_3)_{0.05}$/spiro-OMeTAD/Au	90	17.5	[20]
p-i-n	MgF_2/ITO/PTAA/$MAPbI_3$-NH_4Cl/C_{60}/BCP/Cu	39.5	17.24	[21]
p-i-n	ITO/PTAA/$FA_xCs_{1-x}PbI_3$/C_{60}/BCP/Cu	27.6	20.2	[22]
n-i-p	FTO/SnO_2/$(FAPbI_3)_{0.85}(MAPbBr_3)_{0.15}$/V1366/Au	30.24	19.06	this work

References:

[1] K. Rakstys, S. Paek, A. Drevilkauskaite, H. Kanda, S. Daskeviciute, N. Shibayama, M. Daskeviciene, A. Gruodis, E. Kamarauskas, V. Jankauskas, V. Getautis, M. K. Nazeeruddin, ACS Appl. Mater. Interfaces 2020, 12, 19710.
[2] G.M. Sheldrick, SHELXT - Integrated space-group and crystal-structure determination, Acta Crystallogr. Sect. A Found. Crystallogr. 71 (2015) 3–8. https://doi.org/10.1107/S2053273314026370.
[3] G.M. Sheldrick, Crystal structure refinement with SHELXL, Acta Crystallogr. Sect. C Struct. Chem. 71 (2015) 3–8. https://doi.org/10.1107/S2053229614024218.
[4] O. V. Dolomanov, L.J. Bourhis, R.J. Gildea, J.A.K. Howard, H. Puschmann, OLEX2 : a complete structure solution, refinement and analysis program, J. Appl. Crystallogr. 42 (2009) 339–341. https://doi.org/10.1107/S0021889808042726.
[5] E. Bi, W. Tang, H. Chen, Y. Wang, J. Barbaud, T. Wu, W. Kong, P. Tu, H. Zhu, X. Zeng, J. He, S. ichi Kan, X. Yang, M. Grätzel and L. Han, Joule, 2019, 3, 2748–2760.
[6] T. Bu, X. Liu, Y. Zhou, J. Yi, X. Huang, L. Luo, J. Xiao, Z. Ku, Y. Peng, F. Huang, Y. B. Cheng and J. Zhong, Energy Environ. Sci., 2017, 10, 2509–2515.
[7] H. Chen, F. Ye, W. Tang, J. He, M. Yin, Y. Wang, F. Xie, E. Bi, X. Yang, M. Grätzel and L. Han, Nature, 2017, 550, 92–95.
[8] E. H. Jung, N. J. Jeon, E. Y. Park, C. S. Moon, T. J. Shin, T. Y. Yang, J. H. Noh and J. Seo, Nature, 2019, 567, 511–515.
[9] Z. Liu, L. Qiu, E. J. Juarez-Perez, Z. Hawash, T. Kim, Y. Jiang, Z. Wu, S. R. Raga, L. K. Ono, S. (Frank) Liu and Y. Qi, Nat. Commun., 2018, 9, 1–11.
[10] J. H. Heo, M. H. Lee, M. H. Jang and S. H. Im, J. Mater. Chem. A, 2016, 4, 17636–17642.
[11] J. Li, H. Wang, X. Y. Chin, H. A. Dewi, K. Vergeer, T. W. Goh, J. W. M. Lim, J. H. Lew, K. P. Loh, C. Soci, T. C. Sum, H. J. Bolink, N. Mathews, S. Mhaisalkar and A. Bruno, Joule, 2020, 4, 1035–1053.
[12] S. Pescetelli, A. Agresti, S. Razza, L. A. Castriotta and A. Di Carlo, 2019 Int. Symp. Adv. Electr. Commun. Technol. ISAECT 2019, 2019, 6–10.
[13] G. S. Han, J. Kim, S. Bae, S. Han, Y. J. Kim, O. Y. Gong, P. Lee, M. J. Ko and H. S. Jung, ACS Energy Lett., 2019, 4, 1845–1851.
[14] Y. Deng, X. Zheng, Y. Bai, Q. Wang, J. Zhao and J. Huang, Nat. Energy, 2018, 3, 560–566.
[15] Y. Deng, C. H. van Brackle, X. Dai, J. Zhao, B. Chen and J. Huang, Sci. Adv., 2019, 5, 1–9.
[16] C. H. Chiang, M. K. Nazeeruddin, M. Grätzel and C. G. Wu, Energy Environ. Sci., 2017, 10, 808–817.
[17] A. Agresti, S. Pescetelli, A. L. Palma, B. Martín-García, L. Najafi, S. Bellani, I. Moreels, M. Prato, F. Bonaccorso and A. Di Carlo, ACS Energy Lett., 2019, 4, 1862–1871.
[18] T. Bu, J. Li, F. Zheng, W. Chen, X. Wen, Z. Ku, Y. Peng, J. Zhong, Y. B. Cheng and F. Huang, Nat. Commun., 2018, 9, 1–10.
[19] H. Wang, Z. Huang, S. Xiao, X. Meng, Z. Xing, L. Rao, C. Gong, R. Wu, T. Hu, L. Tan, X. Hu, S. Zhang and Y. Chen, J. Mater. Chem. A, 2021, 9, 5759–5768.
[20] J. Chung, S. S. Shin, K. Hwang, G. Kim, K. W. Kim, D. S. Lee, W. Kim, B. S. Ma, Y. K. Kim, T. S. Kim and J. Seo, Energy Environ. Sci., 2020, 13, 4854–4861.
[21] X. Dai, Y. Deng, C. H. Van Brackle, S. Chen, P. N. Rudd, X. Xiao, Y. Lin, B. Chen and J. Huang, Adv. Energy Mater., , DOI:10.1002/aenm.201903108.
[22] Y. Deng, S. Xu, S. Chen, X. Xiao, J. Zhao and J. Huang, Nat. Energy, 2021, 1–9.
